# Evidence of Partial Migration in a Large Coastal Predator: Opportunistic Foraging and Reproduction as Key Drivers?

**DOI:** 10.1371/journal.pone.0147608

**Published:** 2016-02-03

**Authors:** Mario Espinoza, Michelle R. Heupel, Andrew J. Tobin, Colin A. Simpfendorfer

**Affiliations:** 1 Centre for Sustainable Tropical Fisheries and Aquaculture & College of Marine and Environmental Sciences, James Cook University, Townsville, Queensland, 4811, Australia; 2 AIMS@JCU, Australian Institute of Marine Science, College of Marine and Environmental Sciences, James Cook University, Townsville, Queensland, 4811, Australia; 3 Australian Institute of Marine Science, PMB No 3, Townsville, Queensland, 4810, Australia; Hawaii Pacific University, UNITED STATES

## Abstract

Understanding animal movement decisions that involve migration is critical for evaluating population connectivity, and thus persistence. Recent work on sharks has shown that often only a portion of the adult population will undertake migrations, while the rest may be resident in an area for long periods. Defining the extent to which adult sharks use specific habitats and their migratory behaviour is essential for assessing their risk of exposure to threats such as fishing and habitat degradation. The present study used acoustic telemetry to examine residency patterns and migratory behaviour of adult bull sharks (*Carcharhinus leucas*) along the East coast of Australia. Fifty-six VR2W acoustic receivers were used to monitor the movements of 33 bull sharks in the central Great Barrier Reef (GBR). Both males and females were detected year-round, but their abundance and residency peaked between September and December across years (2012–2014). High individual variability in reef use patterns was apparent, with some individuals leaving the array for long periods, whereas others (36%) exhibited medium (0.20–0.40) or high residency (> 0.50). A large portion of the population (51%) undertook migrations of up to 1,400 km to other coral reefs and/or inshore coastal habitats in Queensland and New South Wales. Most of these individuals (76%) were mature females, and the timing of migrations coincided with the austral summer (Dec-Feb). All migrating individuals (except one) returned to the central GBR, highlighting its importance as a potential foraging ground. Our findings suggest that adult bull sharks appear to be highly dependent on coral reef resources and provide evidence of partial migration, where only a portion of the female population undertook seasonal migrations potentially to give birth. Given that estuarine habitats face constant anthropogenic pressures, understanding partial migration and habitat connectivity of large coastal predators should be a priority for their management.

## Introduction

Large coastal predators are capable of making long-range dispersals across a wide range of habitats. These movements are often driven by seasonal environmental changes [1,2], increasing foraging opportunities [3,4], and reproduction [5,6]. Migration is a specific type of seasonal dispersal characterized by highly directional, long-range movements [7]. Therefore, understanding animal movement decisions that involve migration is critical for evaluating population connectivity, and thus persistence [8,9]. In coastal shark species, movement decisions typically vary throughout ontogeny [10]. Neonate and juvenile sharks, for example, often occupy inshore habitats (e.g. bays and estuaries) with their movement restricted to relatively small areas [11,12]. In contrast, adult sharks generally expand their movements to include offshore habitats due to higher energetic requirements [13]. Mature sharks may also invest more energy in moving longer distances if there is higher success of mating or parturition. Since migration is an energetically demanding activity for animals, the benefits of moving should outweight the cost, and this is often reflected in reproductive success [7].

There is increasing evidence that in many shark species, mature females tend to return to their exact birthplace (natal philopatry) or birth region (regional philopatry) for mating or parturition [14–16]. Moreover, often only a portion of the adult population undertake seasonal reproductive migrations and/or exhibit philopatry, while the rest may be resident to an area for long periods [17–19], a term commonly described in the literature as partial migration [20]. Defining the extent to which large coastal predators use particular habitats and their migratory behaviour is essential for assessing the risk of exposure to threats such as fishing and habitat degradation [21–23]. Ultimately, a better understanding of the spatial ecology of a species can provide important information for defining their role in the ecosystem [24,25], and can help inform managers about effective conservation approaches [26,27].

Quantifying the movement of wide-ranging marine species is often challenging, mainly because of the limitations and trade-offs of current underwater tracking devices [28–30]. Passive acoustic monitoring, for example, provides long-term, low-resolution spatial data (e.g. presence-absence) compared to active tracking or satellite telemetry, and in recent years has become an increasingly popular tool in marine studies. This relatively inexpensive technology has also facilitated the establishment of networks of acoustic receivers through collaborative efforts [31–33], enabling researchers to track long-term movements of single or multiple species simultaneously over broad spatial scales (10–1000 km). Therefore, acoustic receiver networks have increased our understanding of complex behavioural patterns at the population level [18,27,34].

Bull sharks (*Carcharhinus leucas*) are wide-ranging predators known to use both inshore and offshore habitats [12,34,35]. Early life-stages of bull sharks are typically confined to estuarine and riverine systems [12,36,37], while adults tend to use a wider range of habitats along the coast [2,34]. However, our knowledge of the spatial ecology and behavioural patterns of adult bull sharks is still limited. Previous movement studies have suggested that adults were relatively sedentary and confined to nearshore waters [30,38,39], but recent work has revealed complex migratory dynamics and connectivity with offshore coral reef habitats [2,27,34]. Few studies have investigated the use of coral reefs by adult bull sharks, or the drivers involved in movement decisions and habitat connectivity. The present study used several acoustic receiver arrays to examine the residency patterns and migratory behaviour of adult bull sharks monitored in coral reef habitats of the central Great Barrier Reef (GBR). This region, located approximately 70 km off the coast of Townsville, is characterized by the presence of semi-isolated midshelf reefs (5–15 km apart) separated by relatively deep sandy channels (40–70 m). Although these reefs vary in shape and size, they have similar morphologies [40], providing an ideal system to examine how large coastal predators such as bull sharks use coral reef habitats. Specifically, we investigated: (*i*) long-term residency of bull sharks to the central GBR; (*ii*) the influence of biological and environmental drivers on the number of sharks detected and their residency patterns; and (*iii*) the degree of connectivity between reef habitats. Moreover, broad scale movements and migratory dynamics of bull sharks were examined along the East coast of Australia.

## Materials and Methods

### Ethics and fieldwork statement

This research was conducted in accordance with James Cook University animal ethics approval No. A1933. Fieldwork was conducted under Great Barrier Reef Marine Park Authority permit No. G14/36624.1.

### Acoustic array and shark tagging

**Fig 1:**
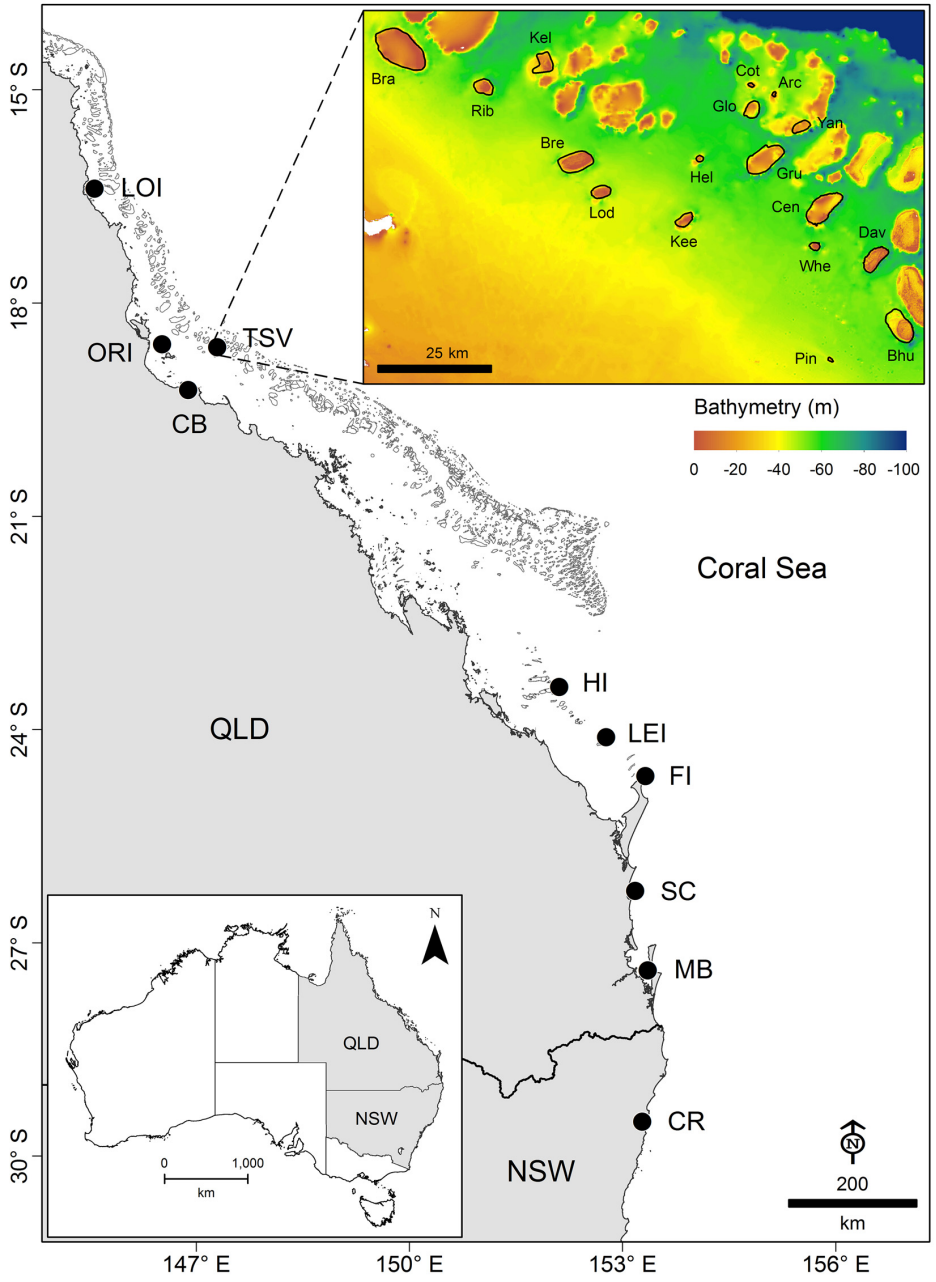
Location of acoustic receiver arrays (black dots) along the East coast of Australia. Receiver arrays: LOI—Low Is.; ORI—Orpheus Is.; TSV—Townsville Reefs; CB—Cleveland Bay; HI—Heron/One Tree Is.; LEI—Lady Elliot Is.; FI—Fraser Is.; SC—Sunshine Coast; MB—Moreton Bay; CR—Clarence River. Inset on the right top corner shows a close-up view of the TSV Reefs: Bra—Bramble; Rib—Rib; Bre—Brewer; Lod—Lodestone; Kee—Keeper; Whe—Wheeler; Broadhurst—Bhu; Dav—Davies; Pin—Pinnacle; Kel—Kelso; Hel—Helix; Gru—Grub; Cen—Centipede; Glo—Glow; Yan—Yankee; Cot—Cotton; Arc—Arc.

An array of 56 Vemco VR2W acoustic receivers (Vemco Ltd, Nova Scotia) was used to monitor shark movements on 17 midshelf reefs from Townsville (TSV), central GBR (Fig 1, S1 Table). Acoustic receivers were deployed along reef slopes at depths between 12 and 20 m. For a detailed description of receiver deployment methodology see [41]. Receiver data were downloaded every 4–6 months between October 2012 and October 2014. Permanent sentinel acoustic transmitters deployed at several locations indicated maximum detection range within the study site which was approximately 350 m (M. Espinoza unpubl. data). Acoustic coverage for each reef was calculated as the total reef area available divided by the sum of detection range areas of the receivers deployed at each reef (S1 Table). This was based on the assumption that each receiver had a maximum detection range of 250 m. Although acoustic coverage is likely to vary at each receiver and between each reef [42,43], this provided an estimate of potential acoustic coverage which ranged from 2.1 to 100% (mean ± SD: 27.1 ± 31.9%).

Nine additional acoustic receiver arrays located in the northern GBR (Low Isles—LOI), central GBR (Cleveland Bay—CB and Orpheus Island—ORI), southern GBR (Lady Elliot Island—LEI and Capricorn Bunker reefs including Heron, Sykes, One Tree Island—HI), southern Queensland (Fraser Island—FI, Sunshine Coast—SC and Moreton Bay—MB) and New South Wales (Clarence River—CR) were used to examine broad scale movement and connectivity of bull sharks along the East coast of Australia (Fig 1). Each receiver array included VR2W acoustic receivers covering habitats as diverse as temperate estuaries, inshore seagrass regions and offshore coral reefs. The number of receivers varied by site: LOI (n = 15), ORI (n = 33), TSV (n = 56), CB (n = 74), HI (n = 50), LEI (n = 6), FI (n = 14), SC (n = 9), MB (n = 29), CR (n = 12), and represented a combined acoustic network of 298 receivers. Receiver arrays were deployed at various times with the earliest (CB) established in 2008, but all were deployed for the entire study period (2012–2014).

Sharks were captured in the TSV array using a variety of fishing methods, including long-lines and drop-lines (see [41] for a description of sampling methodology). Individuals were measured to the nearest cm (fork length—FL), sexed, tagged with an external identification tag and surgically implanted with a Vemco depth-sensing acoustic transmitter (V16P-4H, 69 kHz). Each transmitter emitted a unique identification code and estimated depth in the water column, with a maximum depth rating of 68 m (accuracy ± 3.4 m). Transmitters were programmed on a pseudo-random repeat rate of 50–100 s and had a battery life of approximately 824 d.

### Residency patterns

An individual was considered present in the TSV array when two or more detections (within an hour) were recorded on the same day. A residency index, defined as the number of days individual sharks were detected in the TSV array divided by the number of days monitored (i.e. number of days from the tagging date to the last date of the study period; October 2014), was used to examine shark presence within the array. Since all transmitters were still active at end of the monitoring period, days monitored and days at liberty were the same. The residency index ranged from 0 to 1, where values close to 1 indicated that individuals spent all of their time in the array. A single linkage hierarchical cluster analysis was used to identify individual shark groups based on their residency index to the TSV array. A two sample t-test was used to examine differences in residency between sexes. Simple linear regression was used to examine the relationship between FL and residency. Daily presence metrics were also estimated to assess the level of shark residency to other arrays. These metrics included the mean number of days sharks were detected in each array, the maximum number of days sharks were continuously detected and the residency index. For all metrics, mean values and standard deviations (± SD) were calculated.

Generalized linear models (GLMs) with Gaussian distribution were used to investigate factors that influenced shark presence within the TSV array. Water temperature (temperature—°C), average wind speed (wind—km/h), rain accumulation (rain—mm), sex, month (Jan-Dec) and the interaction between sex and month were the explanatory variables. Environmental data were obtained from the Australian Institute of Marine Science weather stations at Davies, Rib and Kelso reefs (http://data.aims.gov.au/). Daily mean values of environmental data were calculated for the entire array following the methodology of [44] (see S1 Fig). Significant differences in factors and interactions (sex and FL) were evaluated with maximum likelihood ratio tests (χ^2^, p<0.05). Monthly shark residency to the TSV array was examined using mixed effect models. The response variable (monthly residency index) was defined as the proportion of days individuals were detected each month (25 month period ranging from Oct 2012-Oct 2014); therefore, models were fitted with a binomial distribution. Explanatory variables included environmental (temperature, wind and rain) and biological (sex and FL) drivers. To account for unequal sample size of sharks tagged across years and the repeated-measures nature of the data, "Year" and "Tag ID" were treated as random effects in the models [45,46]. Model performance was assessed with AIC_c_ and candidate models (including interaction terms between month, sex and size) compared against a null model: RI ~ 1 + (1 | Tag ID) + (1 | Year). The best fit model was then compared against all candidate models and significant differences were evaluated with maximum likelihood ratio tests (χ^2^, p<0.05). Models were tested for multicollinearity using the “*VIF*” function in the *AED* package [46] and by examining pairwise correlation plots between predictors. Mixed effect models were implemented using the “*glmer*” function from the “*lme4*” library [47] in R v.3.0.2 [48].

### Inter-reef movements and habitat connectivity

Shark movements were examined using centre-of-activity (COA) to calculate mean position (latitude and longitude) from hourly detection data weighted by the number of detections at each receiver [49]. Position data were converted to Universal Transverse Mercator Projection (UTM Zone 55; m) and minimum linear dispersal (i.e., linear distance travelled between two positions; km) and minimum dispersal time (i.e., difference in time travelled between two positions; hr) were calculated. A roaming index (i.e. proportion of reefs visited relative to the total number of reefs monitored) was used to examine the extent of shark movement within the TSV array. Roaming index ranged from 0 to 1, where values close to 1 indicated an individual was detected on all monitored reefs. A two sample t-test was used to examine differences in roaming between sexes. Simple linear regression was used to examine the relationship between FL and roaming index. To quantify the degree of reef connectivity within the TSV array, we constructed a movement matrix of individual sharks moving from/to each reef. In this matrix, the total number of individuals detected for the entire monitoring period was combined at the reef level. A modified circular plot (“connectivity plot”) was used to visualize the number of incoming and outgoing movements of male and female bull sharks within the TSV array. The degree of shark connectivity between TSV and other arrays was also investigated using this approach. Connectivity plots were implemented using the "*circos*.*trackPlotRegion*" function from the "circlize" package [50] in R v.3.0.2 [48].

## Results

### Shark presence and residency to the TSV array

**Fig 2:**
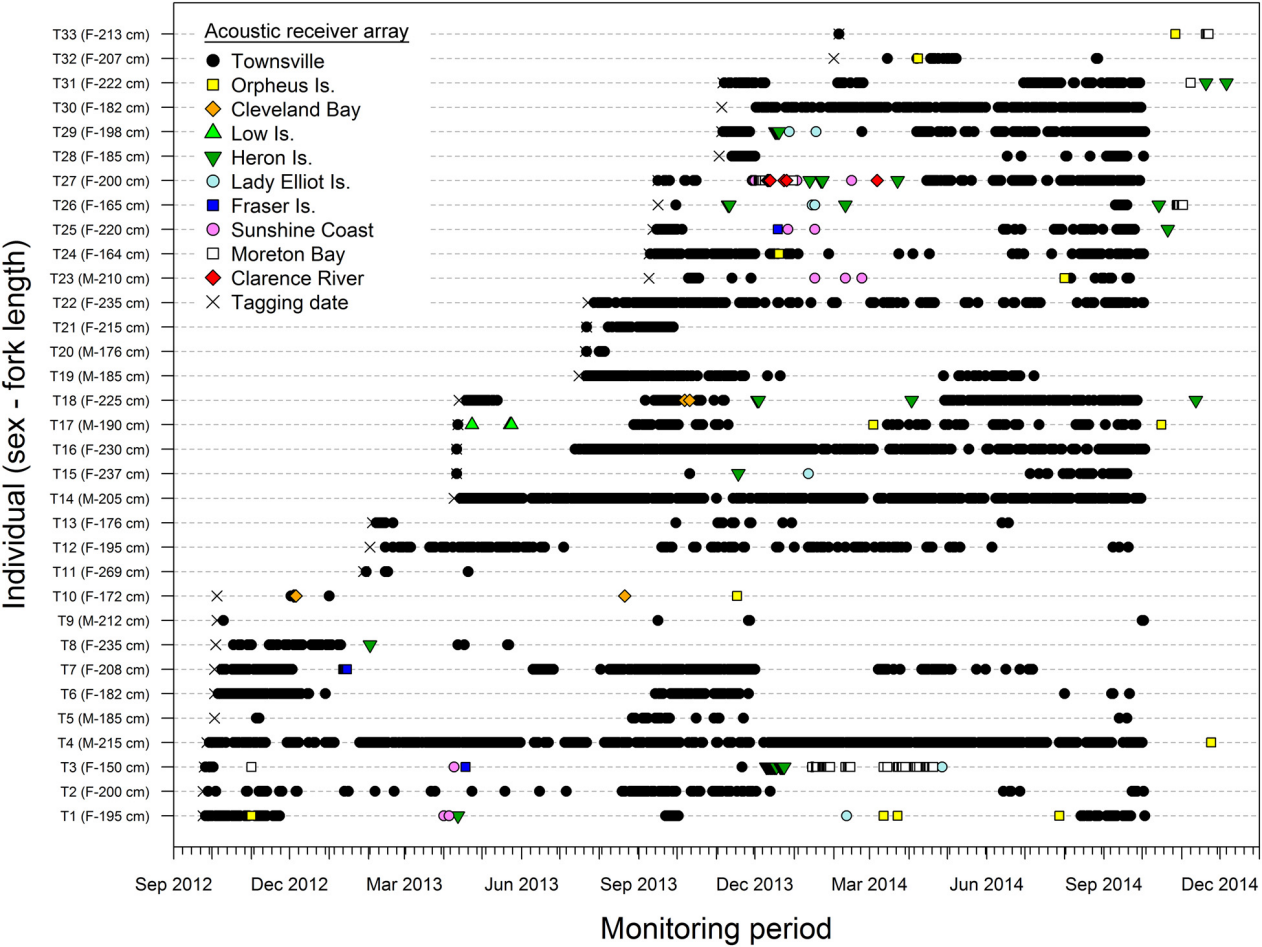
Presence plot of bull sharks (*Carcharhinus leucas*) monitored along the East coast of Australia. The tag number, sex (M—males; F—females) and size (fork length) of individual sharks are provided.

Thirty-three bull sharks were acoustically tagged and monitored in the central GBR between February 2012 and October 2014. Size of bull sharks ranged from 150–269 cm FL (mean ± SD: FL = 201.8 ± 25.2 cm), with a F:M ratio of 25:8 (Table 1). All sharks were classified as adults or sub-adults based on their size and the calcification of male claspers. Most individuals were tagged at Lodestone (n = 12), John Brewer (n = 6) and Rib (n = 4) reefs; the rest (33%) were tagged opportunistically at seven other reefs within the array (Table 1). One mature female (T33), monitored for 237 d, was only detected in the TSV array once, but was detected 10 months later in ORI and MB (Fig 2). This individual was excluded from the estimation of residency index as it likely left the TSV array on the day of tagging. The remaining 32 bull sharks were monitored from 241–737 d and detected for at least 3 d, remaining on average 95 ± 108 d in the array (Table 1).

**Table 1:** Bull shark (*Carcharhinus leucas*) residency to the central Great Barrier Reef (GBR). Sex: M—males; F—females. FL: fork length. DM—number of days monitored; DD—number of days detected; RI—residency index (proportion of days detected); RoI—roaming index (proportion of reefs visited); M—migrating individual detected at other receiver array; R—migrating individual that returned to the central GBR.

TagID	Tagging Reef	Sex	FL (cm)	Tagging Date	DM	DD	Max. DD	Reefs Visited	Migrating Event	RoI	RI
T1	Rib	F	195	8/10/12	737	73	11	7	M-R	0.41	0.10
T2	Rib	F	200	8/10/12	737	83	8	5		0.29	0.11
T3	Rib	F	150	9/10/12	736	6	4	3	M-R	0.18	0.01
T4	Lodestone	M	215	11/10/12	734	473	24	11	M	0.65	0.64
T5	John Brewer	M	185	17/10/12	728	22	4	4		0.24	0.03
T6	John Brewer	F	182	17/10/12	728	107	12	5		0.29	0.15
T7	John Brewer	F	208	17/10/12	728	188	15	13		0.76	0.26
T8	John Brewer	F	235	18/10/12	727	41	5	9	M-R	0.53	0.06
T9	John Brewer	M	212	19/10/12	726	4	2	5		0.29	0.01
T10	John Brewer	F	172	19/10/12	726	3	2	4	M-R	0.24	<0.01
T11	Keeper	F	269	11/02/13	611	5	3	5		0.29	0.01
T12	Rib	F	195	16/02/13	606	116	7	4		0.24	0.19
T13	Lodestone	F	176	18/02/13	604	24	8	3		0.18	0.04
T14	Wheeler	M	205	23/04/13	540	374	29	4		0.24	0.69
T15	Helix	F	237	25/04/13	538	34	15	7	M-R	0.41	0.06
T16	Helix	F	230	25/04/13	538	274	21	10		0.59	0.51
T17	Lodestone	M	190	26/04/13	537	80	8	9	M-R	0.53	0.15
T18	Lodestone	F	225	27/04/13	536	139	18	10	M-R	0.59	0.26
T19	Centipede	M	185	30/07/13	442	113	25	8		0.47	0.26
T20	Lodestone	M	176	4/08/13	437	6	5	4		0.24	0.01
T21	Lodestone	F	215	5/08/13	436	41	14	8		0.47	0.09
T22	Lodestone	F	235	6/08/13	435	150	14	11		0.65	0.34
T23	Bramble	M	210	23/09/13	387	18	4	4	M-R	0.24	0.05
T24	Bramble	F	164	23/09/13	387	111	15	6	M-R	0.35	0.29
T25	Keeper	F	220	26/09/13	384	50	15	10		0.59	0.13
T26	Davies	F	165	30/09/13	380	11	10	5	M-R	0.29	0.03
T27	Broadhurst	F	200	30/09/13	380	91	10	7	M-R	0.41	0.24
T28	Lodestone	F	185	17/11/13	332	39	14	5		0.29	0.12
T29	Lodestone	F	198	19/11/13	330	123	54	7	M-R	0.41	0.37
T30	Lodestone	F	182	19/11/13	330	170	11	11		0.65	0.52
T31	Lodestone	F	222	20/11/13	329	76	7	12	M	0.71	0.23
T32	Helix	F	207	15/02/14	242	14	2	8	M-R	0.47	0.06
T33	Lodestone	F	213	19/02/14	238	1	1	2	M	0.12	<0.01

**Fig 3:**
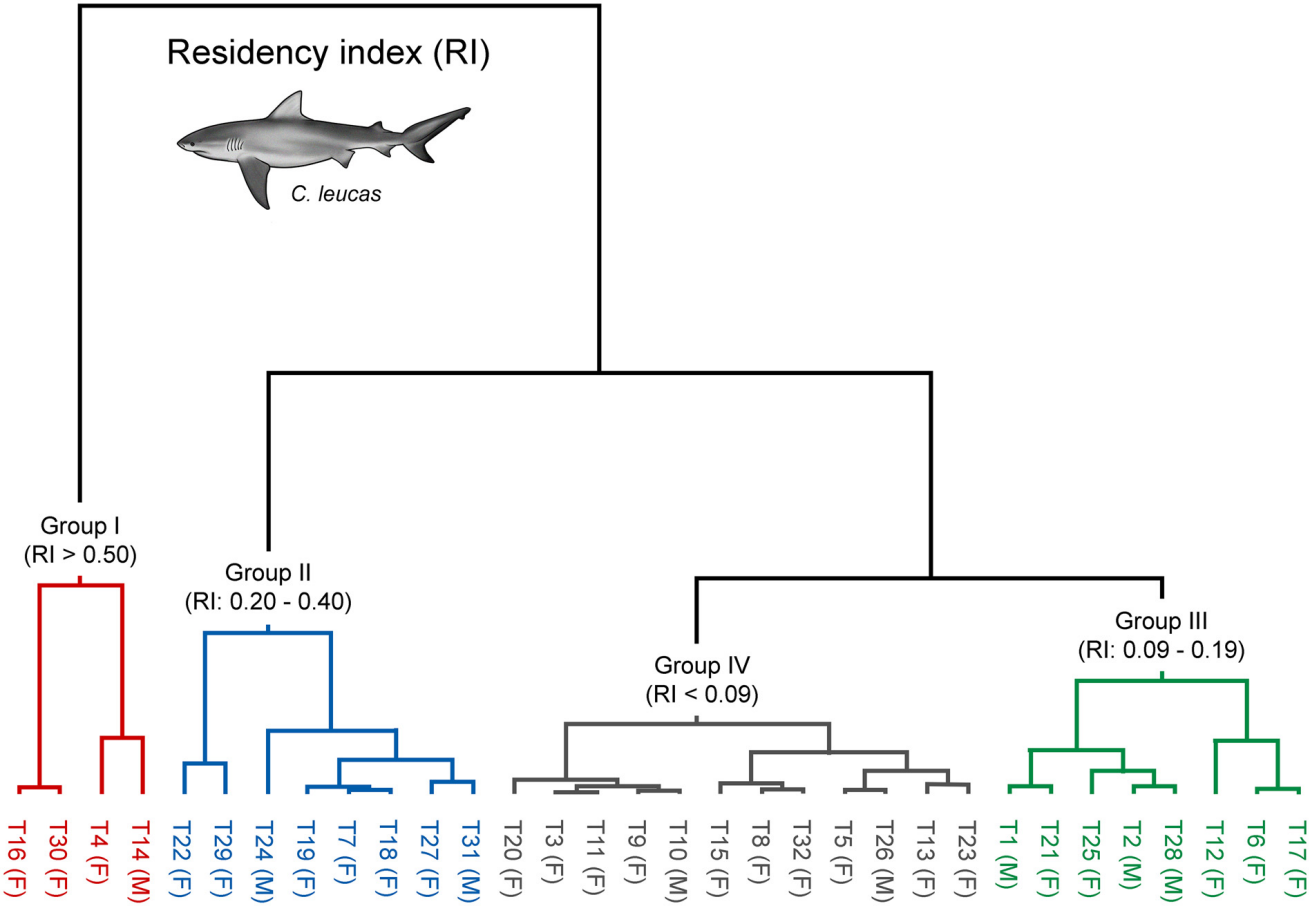
Dendrogram showing individual bull sharks (*Carcharhinus leucas*) grouped based on their residency to the Townsville Reefs.

The maximum number of days sharks were continuously detected in the TSV array ranged from 2–54 d (12 ± 10 d). Residency of bull sharks to the array ranged from 0.004–0.69 (0.19 ± 0.19), and did not differ between males (0.23 ± 0.28) and females (0.17 ± 0.15; t-test = 2.04, df = 30, p = 0.421). There was no relationship between residency and FL (t-test = 0.775, p = 0.445); however, cluster analysis identified four main groups of sharks based on their residency index (Fig 3). Group I included four individuals that exhibited high residency to the TSV array (0.51–0.69) and were continuously detected for up to 29 days (Table 1, Fig 3). Group II included eight individuals with intermediate residency (0.20–0.40). Eight sharks in group III and twelve sharks in group IV had either a low (0.09–0.19) or very low (< 0.09) residency index to the TSV array, respectively. There was no clear pattern of group separation by size or sex; a similar portion of the tagged population of males and females were classified as having low (< 0.20) or high residency (> 0.20) to the TSV array (Fisher's Exact Test, p > 0.05). Sharks in groups III and IV were absent 527 ± 161 d of the total days monitored. Interestingly, except for two individuals (male T20 and female T21), remaining bull sharks in groups III and IV either left TSV and came back after a few weeks/months or did not come back but were detected in other arrays.

**Fig 4:**
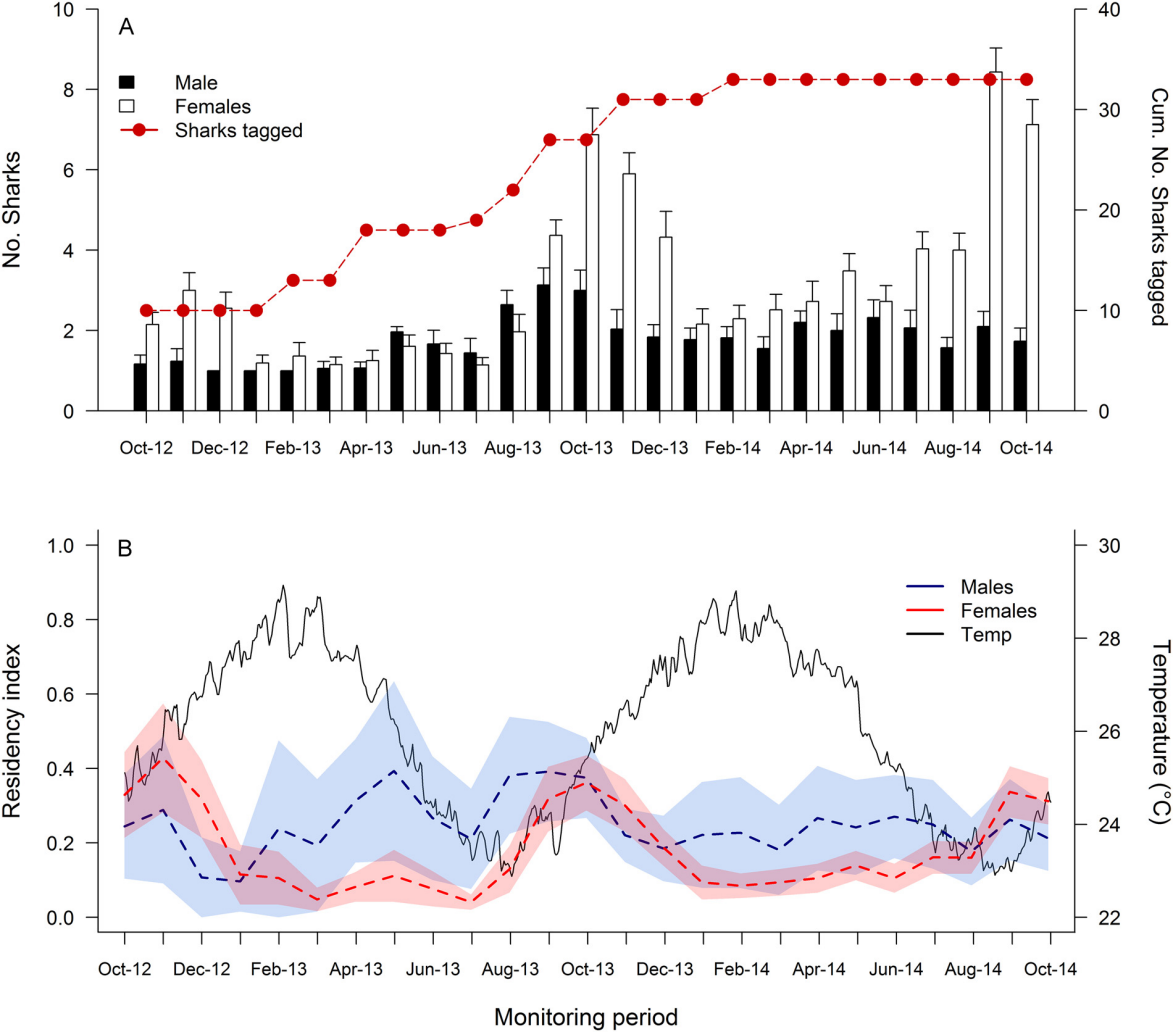
Pattern of occurrences of bull sharks (*Carcharhinus leucas*) monitored in the central Great Barrier Reef. Panels show: (A) mean number of sharks detected (± SE) and cumulative number of sharks tagged during the monitoring period; (B) mean residency index (± 95% confidence intervals—shaded polygons) and temperature fluctuations recorded during the monitoring period.

The number of sharks detected within the TSV array fluctuated throughout the study period, with the greatest number of sharks recorded between September and December across years (Fig 4A). Males and females showed similar patterns of presence in the TSV array, but females were more abundant than males during the spring (Sep-Nov; Table 2, S2A Fig). Females generally left the study site late in the spring or early in the summer, but most of them returned to the array after a few weeks or months (Fig 2). During this period, a large number of females were detected in inshore arrays, including CB, ORI, SC, MB and CR (Figs 1 and 2). Temperature and wind also influenced the number of sharks detected within the TSV array (Table 2), with greater numbers of sharks recorded at higher water temperatures (S2B Fig) and lower wind speeds (S2C Fig). Interestingly, some females seem to leave the TSV array at water temperatures between 24–25°C (Fig 4B). Results from mixed effect models were driven by seasonality of female bull sharks (e.g., monthly presence of females) within the TSV array. Males on the other hand seemed to be detected year-round (Fig 4B). Twenty-six of 29 candidate models were significantly better than the null model (S2 Table). The top two candidate models (ranked based on their AIC_c_) included interactions between month × sex (AIC_c_ = 4651.8) and month × FL (AIC_c_ 4726.7), which were statistically similar but differed from the rest (S2 Table). Models with rain, wind and temperature ranked among the top ten, but explained a lower proportion of shark residency than models that included month.

**Table 2:** General linear model results of factors that influence the number of bull sharks (*Carcharhinus leucas*) detected in the central Great Barrier Reef. Explanatory variables included month, sex, water temperature (Temp—°C), average wind speed (Wind—km/h) and rain accumulation (Rain—mm). Significant differences were evaluated with maximum likelihood ratio tests (χ2, p<0.05).

**Factor**	**df**	**Deviance**	**Resid Dev**	**p-value**
Null			9673.4	
Month	11	2328.7	7344.6	<0.001
Sex	1	2725.6	4619.0	<0.001
Temp	1	41.1	4577.9	<0.001
Wind	1	45.7	4532.2	<0.001
Rain	1	0.3	4531.8	0.5586
Month × Sex	11	400.8	4131.1	<0.001

### Inter-reef movements and habitat connectivity

**Fig 5:**
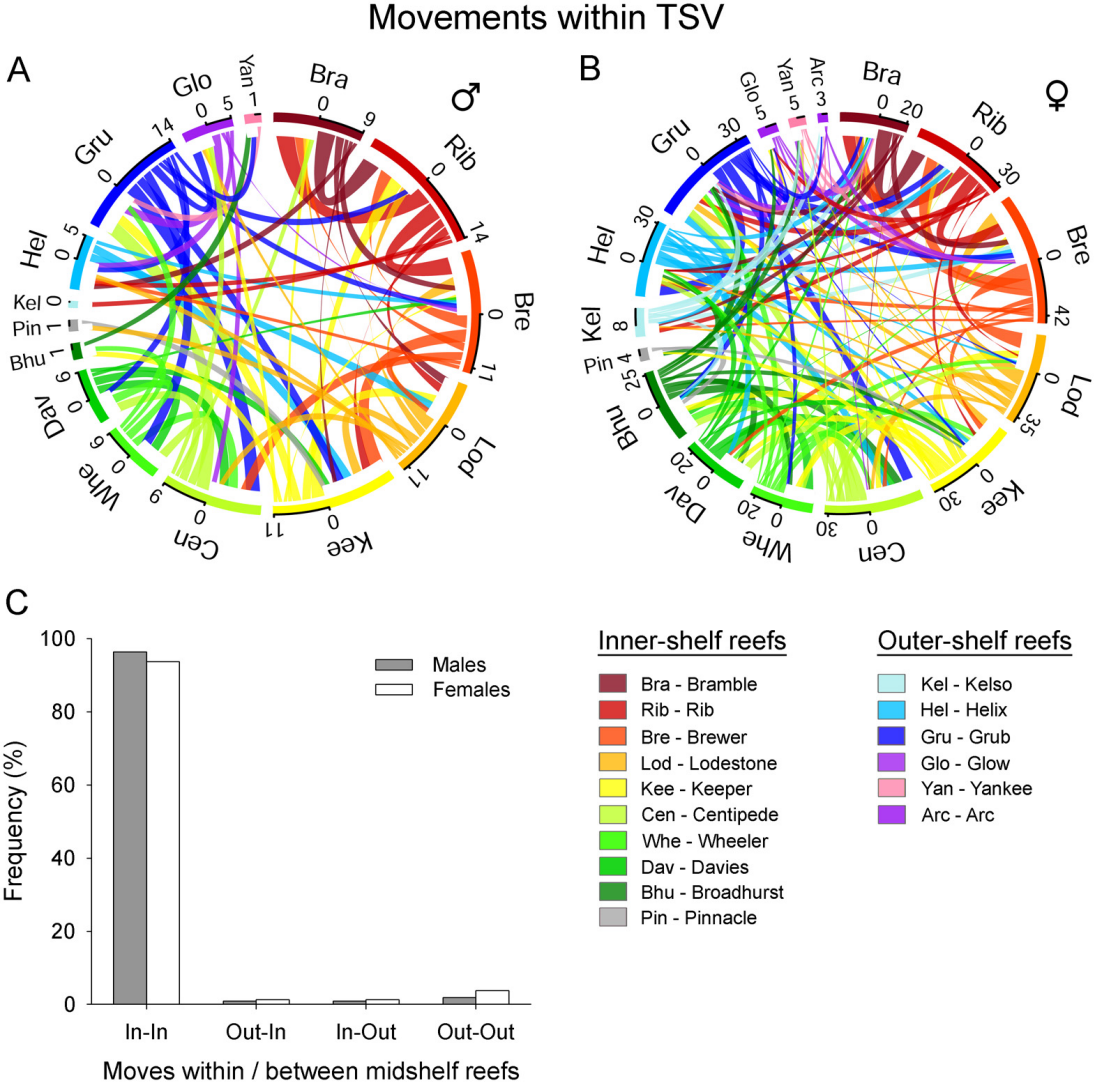
Connectivity plot of bull sharks (*Carcharhinus leucas*) monitored in the central Great Barrier Reef. Panels A and B show the number of male and female bull sharks moving between monitoring reefs. Each colour indicates a unique reef and arrows represent the number of individual sharks that move between reefs. Panel C shows the frequency of moves (%) within and between inner and outer midshelf reefs.

All individuals monitored in the TSV array moved beyond their tagging reef, revealing complex patterns of inter-reef connectivity (Fig 5A and 5B, S1 Video). Connectivity plots from male (Fig 5A) and female (Fig 5B) bull sharks showed high degree of connectivity between offshore reefs, indicating that this species tend to utilize a large number of reefs within the TSV array. Although inter-reef movements were complex, the frequency and magnitude of sharks moving in this reef system reflected a more coastal movement strategy. For instances, more sharks were recorded moving to inner midshelf reefs (e.g., Brewer, Lodestone, Keeper, Rib and Bramble) than across the shelf. Moreover, the frequency of shark movements within inner midshelf reefs was considerably larger than movements across the outer midshelf region (Fig 5C), indicating that adult bull sharks generally spend more time at offshore reefs that were closer to shore. The number of reefs visited by bull sharks varied between 3 and 13 (Table 1). There was no relationship between the number of reefs visited and monitoring days (F_1, 31_ = 0.31, p = 0.579). Roaming index ranged from 0.12 to 0.76 (0.40 ± 0.17), indicating that most individuals used a large proportion of the monitoring reefs within the TSV array. Roaming did not differ between males (0.36 ± 0.16) and females (0.42 ± 0.18; t-test = 2.04, df = 31, p = 0.437), but there was a positive relationship between FL and roaming (F_1, 31_ = 6.37, p = 0.017; R^2^ = 0.17) with larger individuals using a greater number of monitoring reefs than smaller sharks.

**Fig 6:**
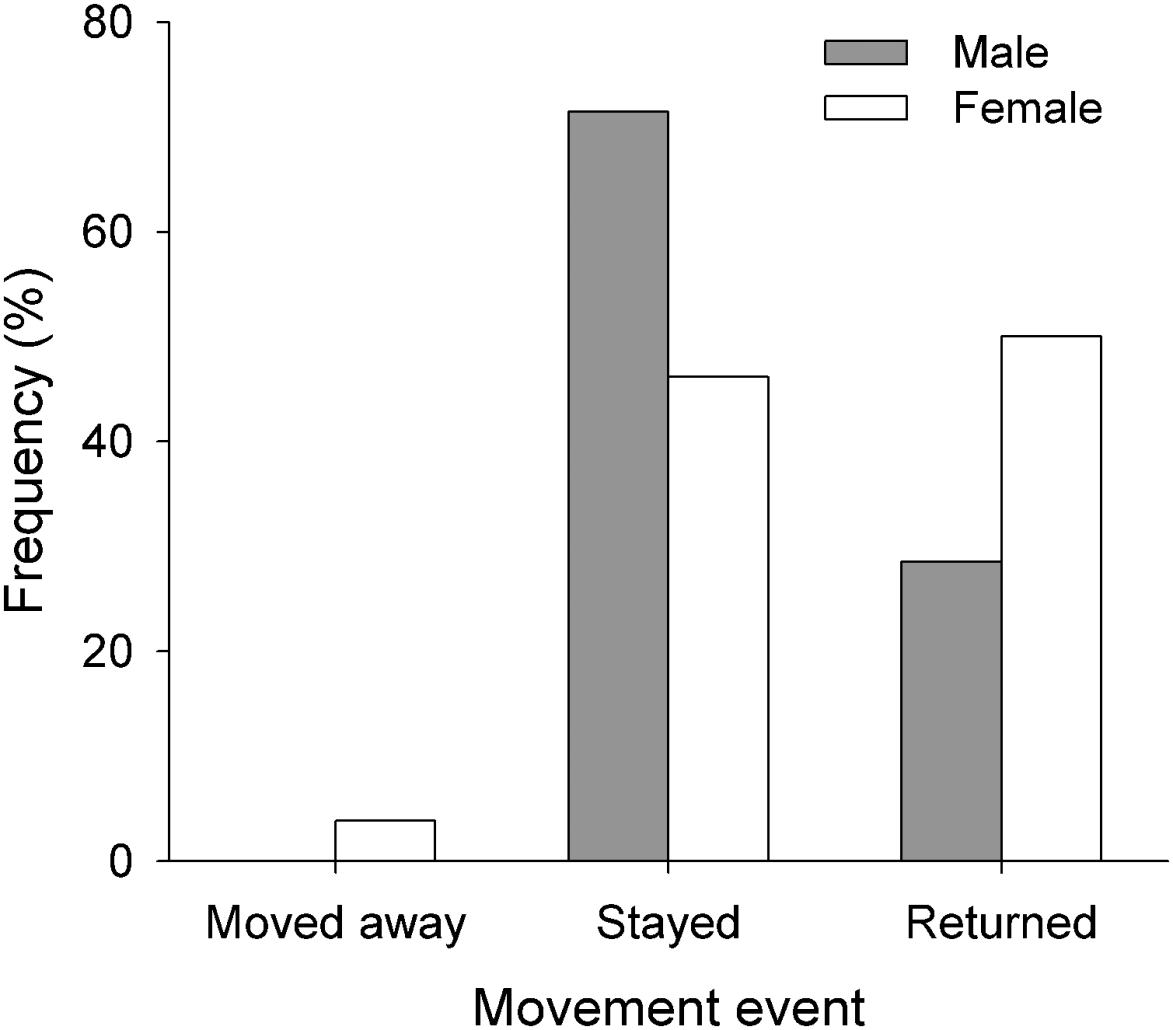
Proportion of bull sharks (*Carcharhinus leucas*) that: (i) moved away from TSV and was detected in other arrays; (ii) stayed and/or was only detected within TSV; and (iii) returned to TSV.

**Fig 7:**
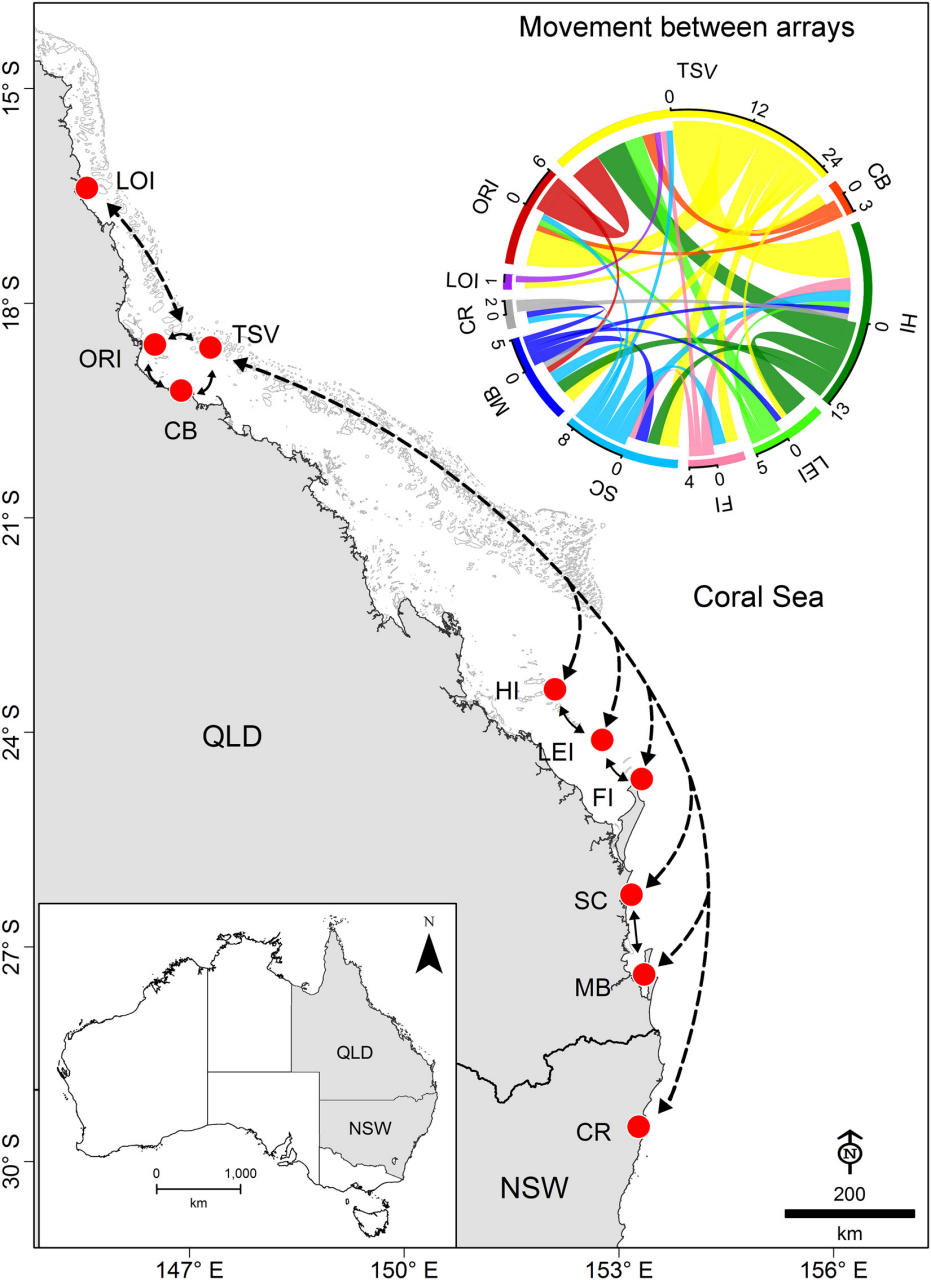
Migratory patterns and broad scale connectivity of bull sharks (*Carcharhinus leucas*) in Queensland (QLD) and New South Wales (NSW), East coast of Australia. Acoustic receiver arrays are indicated by red dots and dashed lines represent movement path between arrays. Each colour from the connectivity plot indicates a unique array and arrows represent the number of individual sharks that move between arrays. Receiver arrays: LOI—Low Is.; ORI—Orpheus Is.; TSV—Townsville Reefs; CB—Cleveland Bay; HI—Heron/One Tree Is.; LEI—Lady Elliot Is.; FI—Fraser Is.; SC—Sunshine Coast; MB—Moreton Bay; CR—Clarence River.

An examination of the broad scale movement and connectivity of bull sharks along the East coast of Australia revealed that more than half of the population (n = 17) was detected in other arrays (Table 3, Figs 6 and 7). Most of these individuals made returned trips to TSV; few individuals moved away from TSV and were not detected at other arrays (Fig 6). Overall, bull sharks exhibited high degree of connectivity along the coast, with most individuals moving from TSV to HI and ORI. Movements between TSV and CB were uncommon despite its proximity (Fig 7). Moreover, individuals detected at HI generally moved to other arrays in southern Queensland (e.g., LEI, FI, SC and MB). Only two of them moved to CR in NSW, indicating that most bull sharks tagged in the central GBR remained within QLD waters (Fig 7).

**Table 3:** Summary information of bull sharks (*Carcharhinus leucas*) monitored along the East coast of Australia. Receiver arrays are ordered based on their geographic location. Values indicate mean ± SD. Receiver arrays: LOI—Low Is; ORI—Orpheus Is.; TSV—Townsville Reefs; CB—Cleveland Bay; HI—Heron/One Tree Is.; LEI—Lady Elliot Is.; FI—Fraser Is.; SC—Sunshine Coast; MB—Moreton Bay; and CR—Clarence River.

Receiver Array	No. Receivers	No. Sharks	No. Days Detected	Distance from TSV (km)	Nearest distance to Shore (km)	Time travelled from TSV (d)
LOI	15	1	3	310	15	11-96 (53 ± 61)
ORI	33	8	2 ± 1	78	16	1-262 (31 ± 77)
TSV	56	33	96 ± 108	-	65	-
CB	74	2	3 ± 1	79	6	1-26 (10 ± 11)
HI	50	11	3 ± 4	730	65	19-521 (100 ± 137)
LEI	6	6	1 ± 1	833	78	31-173 (93 ± 71)
FI	14	3	2 ± 2	912	56	20-147 (69 ± 68)
SC	9	5	3 ± 1	1038	14	44-147 (72 ± 50)
MB	29	5	9 ± 13	1161	1.5	30-40 (35 ± 7)
CR	12	1	6	1400	< 1	*

* Time travelled to CR was not calculated because movements originated from HI and MB.

Female bull sharks undertook longer excursions than males. Only four males were detected in arrays other than TSV such as ORI (central GBR); however, two males (T17 and T23) made returned trips to LOI (northern GBR) and SC (southern Queensland), respectively. Migrating females were detected on all of the arrays, except for LOI (S2 Video). On average, males and females spent < 5 d at other arrays (Table 3). One individual (T3), however, was detected for up to 9 consecutive days at HI (14 d in total) and 7 consecutive days at MB (30 d in total). A large proportion of migrating females (85%) were also detected on inshore arrays. While some of these arrays were relatively close to TSV (e.g. CB and ORI; < 80 km), others, including SC, MB and CR, were over 1,000 km away (S3 Fig). Females travelling south of the GBR were typically detected at HI, LEI and FI prior to detection further south. With the exception of one individual (T33) that was only detected in TSV during its tagging date, all of the sharks that undertook long-range dispersal during the monitoring period (October 2012-October 2014) eventually returned to TSV (Fig 6). Additional data revealed that individual T33 was detected at ORI and MB after November 2014. Travel time of migrating individuals ranged from 1–521 d. Most individuals spent less time travelling between TSV and some of the closest arrays (CB and ORI), but there was no relationship between array proximity and average travel time (F_1, 5_ = 1.632, p = 0.257; Table 3). One individual (T27), for example, travelled 1,400 km in 38 d, while for others (T1, T17 and T23) there were over 150 d between detection at TSV and ORI.

**Fig 8:**
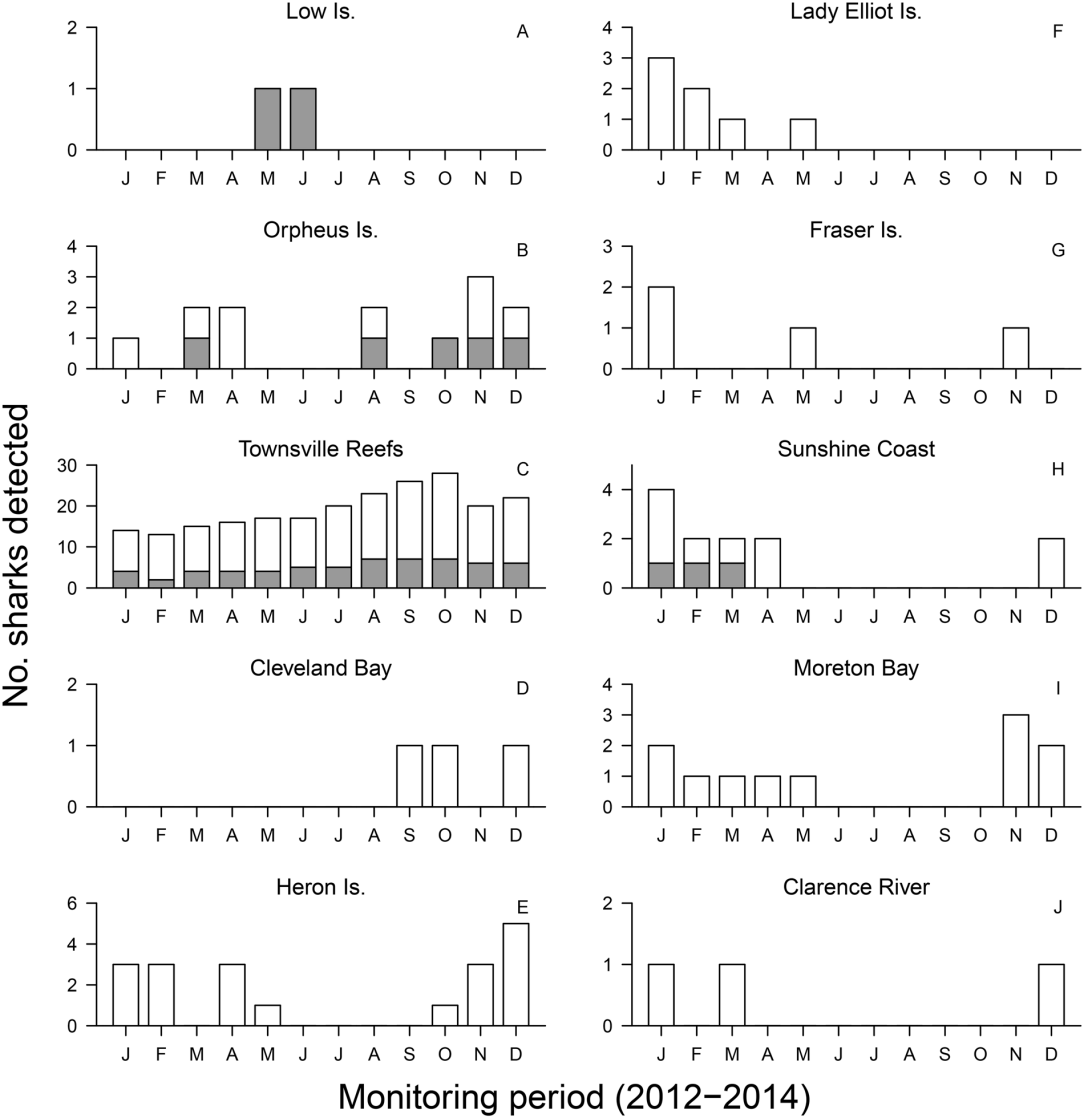
Number of bull sharks (*Carcharhinus leucas*) detected in different acoustic receiver arrays along the East Coast of Australia (grey bars—males; white bars—females).

Monthly patterns of shark presence varied considerably among arrays (Fig 8). In TSV, males and females were present year-round, but in other arrays individuals were mainly detected during summer months (Dec-Feb). These patterns were more evident on nearshore arrays such as ORI, CB, SC, MB and CR. Interestingly, even in these coastal arrays females showed different patterns of depth use in specific months. For example, the only female detected in CR (T27; 200 cm FL) used water depths less than 10 m during December and January, and moved to deeper water (~30 m) in March. In CB, females moved from average depths of 5 m during September and October to less than 3 m in December. In contrast, in MB females were detected at depths of 10–15 m during December and January, and shallower (~5 m) between February and March. With the exception of LOI, at offshore coral reef sites (HI, LEI and FI) sharks were detected between October and May, possibly as they moved to or returned from inshore habitats. Male T17 was the only individual detected in the northern GBR, the rest of the bull shark population was generally detected within the central and southern GBR.

## Discussion

This study demonstrated that coral reefs are important habitats for adult bull sharks, and highlights the large geographic scale of movements and connectivity in this marine predator. Both males and females monitored in reef habitats from the central GBR were detected year-round, but their abundance peaked between September and December across years (2012–2014). There was, however, high individual variability in reef use patterns, with some individuals leaving the area for long periods, while others (36%) exhibited medium (0.20–0.40) or high residency (> 0.50). A large portion of the population (52%) undertook long-range migrations of up to 1,400 km along the East coast of Australia. Most of these migrating individuals were females, and the timing coincided with the austral spring and early summer. An increase in detections at offshore coral reefs from the southern GBR (e.g., Capricorn Bunkers group) and inshore coastal habitats from southern Queensland and New South Wales during this period (late Spring and summer) suggest that females may be moving south for parturition [51]. Moreover, all migrating individuals, except for one, returned to the central GBR after a few weeks or months, highlighting its importance as a potential foraging ground. Collectively, these results provide evidence of partial migration, indicating that only a portion of the bull shark population stay in the central GBR for long periods, whereas the remaining individuals (mainly females) undertake seasonal migrations, potentially to give birth.

Despite the increasing number of studies on reef-associated sharks over the past 10 years, there is still limited knowledge about how large, wide-ranging predators use coral reef habitats [2,18,34,52]. Our data showed that residency of bull sharks to the central GBR was variable, but some individuals remained for long periods. These results are consistent with previous studies of adult bull sharks at Pinnacle Reef in southern Mozambique [2], the Shark Reef Marine Reserve in Fiji [53] and in southern Florida waters [39]. However, bull sharks monitored in Fiji exhibited considerably higher residency (mean = 0.69) than in Mozambique (mean = 0.14) and TSV (mean = 0.19). This could have been due to the fact that: (i) 75% of individuals acoustically tagged at Fiji were monitored for less than 70 d while individuals from other locations were monitored for up to 486 d; (ii) Fiji is an isolated archipelago where shark dispersal may be limited compared to coral reefs within continental shelves [2,34]; and (iii) the Shark Reef Marine Reserve in Fiji is a food provisioning site that attracts bull sharks [53]. Although [53] showed that some bull sharks were absent from the study site for periods of weeks to months before returning, it is unclear how food provisioning sites may influence long-term residency patterns of coastal predators. In our study, bull sharks were monitored for up to two years and exhibited similar residency and migratory patterns to those reported by [2]. Consequently, the behaviours reported for the GBR are likely to be representative of bull shark populations in other coral reefs along continental shelves.

Bull sharks showed a distinct seasonal pattern in presence in the central GBR. In addition, the number of sharks detected in the central GBR fluctuated throughout the study period, with the greatest number of sharks recorded between September and December across years. Seasonal patterns of abundance and residency have been documented in a wide range of reef-associated species, including tiger (*Galeoccerdo cuvier*) [18,54], scalloped hammerhead (*Sphyrna lewini*) [55], silvertip (*C*. *albimarginatus*) [44], and bull sharks [2,39,53], but the drivers influencing those patterns often differ between species and/or geographic regions. In southern Florida, for example, adult bull sharks are present year round in shallow coastal habitats, but catch rates are generally higher during winter months [39]. In contrast, in Mozambique, bull sharks are known to exhibit prolonged periods of residency to offshore reef habitats during summer months, and subsequently detected at inshore locations along the southern East coast of Africa [2]. Based on their findings, [2] suggested water temperature could be a major driver influencing seasonal patterns of occurrence, whereas in southern Florida the presence of large bull sharks coincided with peak tarpon abundance [39]. Our data indicate that water temperature and wind speed also influenced the number of individuals detected. As expected, an increase in wind speed negatively affected detectability of bull sharks within the array (i.e. acoustic detection range may be susceptible to disturbances caused by wind), whereas the number of sharks detected generally increased with increasing temperature. These results are consistent with previous studies that examined the role of environmental drivers on reef shark presence [41,56,57]. However, despite the fact that our study site and Mozambique are located at similar latitudes, and experience a similar range of temperatures, bull sharks monitored at these two locations exhibited different movement patterns [2]. This suggests that although temperature was an important factor in our study, seasonal patterns in reef use may be attributed to biological requirements such as reproduction and foraging [18,39,53,58].

Bull sharks were more abundant and had higher residency to the central GBR in the spring than in the summer. Interestingly, these patterns were mainly driven by the behaviour of females. While some individuals were detected in the central GBR year-round, a large portion of female population left the study site late in the spring/early in the summer and undertook long-range migrations. During this period, migrating females were often detected in coastal arrays of Queensland (CB, ORI, FI, SC and MB) and New South Wales (CR); the rest were detected at offshore reefs (HI and LEI), presumably on their way to or from similar inshore habitats along the coast. Partial migration is known to occur in many teleost species [20,59], but has been poorly studied in elasmobranch fishes [18,19]. In tiger sharks, for example, a recent study revealed that a portion of the population of females moved from remote French Frigate Shoals atoll to the Main Hawaiian Island (MHI) during late summer/early fall, potentially to give birth [18]. Recent studies also seem to indicate that bull sharks monitored in southern Africa and eastern Australia undertake regular coastal migrations, but contrary to our study these migration events have been observed in both sexes and/or tropical and temperate populations exhibited different movement dynamics [2,34]. Bull sharks have a gestation period of approximately 10–11 months, with pupping occurring in late spring/summer and a biennial reproductive cycle [60]. The fact that only a portion of the female population tagged in TSV was detected in nearshore arrays, that visits were relatively short in duration (< 5 d on average), and that the timing of these events coincided with parturition reported for this species suggest that: (i) inshore movements were potentially to give birth; (ii) female reproductive migrations may be a major driver influencing seasonal patterns of reef use and population structure along the East coast of Australia; and (iii) females may return to their birth place and/or birth region for parturition. Reproductive philopatry has been reported for several Carcharhinid sharks [14–17]. A recent study using mitochondrial DNA, for example, showed population structure between juvenile bull sharks residing in closely-spaced nurseries of northern Australia, which suggest that mature females may use specific nurseries in multiple breeding events [61]. Evidence of reproductive philopatry and biennial cycles in bull sharks could have explained why some females moved greater distances while others appeared more resident within the central GBR. Alternatively, females may be moving to closer inshore areas for parturition rather than undertaking long-range migrations. Our findings highlight the need to better understand complex movement decisions and migratory behaviour of large marine predators, especially when they only involve a portion of the adult population.

This study not only demonstrated that bull sharks are capable of substantial migrations, but also showed that a large fraction of the population that left the central GBR (>85%) eventually returned. Similar findings have been reported for other bull shark populations in both tropical [2] and temperate waters [34], which raises an important question: why do bull sharks, particularly individuals that undertook long-range migrations, return to the central GBR? As previously discussed, migrations to inshore coastal arrays may be attributed to parturition and philopatry [17]. However, returning to reefs within the central GBR could be driven by seasonal foraging opportunities [18,53,54]. Our data indicated that a larger number of bull sharks were generally present in the central GBR during annual spawning aggregations (September-November) of Spanish mackerel (*Scomberomorus commerson*) [62]. Spanish mackerel are known to form large predictable aggregations in some of the midshelf reefs from the central GBR (e.g. Bramble, Rib, John Brewer, Helix and Lodestone) during their spawning (A. Tobin unpublished data), which could explain the presence of bull sharks during this period. There is increasing evidence that bull sharks spend a disproportionate amount of time at these same reefs where large aggregations of Spanish mackerel are known to occur and/or use movement corridors along inner midshelf reefs within this region [27], potentially as foraging strategy. These findings suggest that the central GBR may be an important foraging ground for bull sharks, particularly during Spanish mackerel spawning periods. Previous work on tiger and bull sharks have also shown that some individuals return to specifc areas to forage on seasonaly abundant resources [18,52,53].

Other foraging strategies could also be driving the movement and seasonal patterns of bull sharks to the central GBR. Historically, this region has been an important ground for Queensland fisheries targeting coral trout (*Plectropumus* spp.), redtroat emperor (*Lethrinus miniatus*) and Spanish mackerel aggregations [62–64]. Therefore, it is unclear whether bull sharks prey directly on common resources that tend to aggregate or whether they are attracted to this area because it is a common fishing ground where they could potentially get an “easy meal”. In Fiji, for example, bull sharks are attracted to a food provisioning site on both feeding or non-feeding days, but they tended to remain longer when feeding takes place [53]. Interestingly, [53] found that sharks tended to leave the study site for longer periods during the summer, before returning to the feeding site again. Therefore, even at food provisioning sites, reproduction may be involved in their migratory dynamics. Bull sharks have also been observed preying on tarpon during recreational catch and release angling [65], thus supporting our hypothesis that fisheries operating in the central GBR may influence their seasonal patterns. This could mean that even if spawning aggregations also occur in other areas of the GBR, bull sharks may move to or return to TSV because they may learn to recognize these grounds as potential sources of food. Future studies, however, should investigate in more detail the spatial and temporal overlap between bull sharks and reef fisheries operating in the GBR.

Acoustic telemetry studies are often limited by acoustic coverage and/or behaviour of aquatic animals [28,29]. For example, wide-ranging species can have long periods of absence or remain outside the detection range of the receivers even if animals are within the study site [66]. Proper consideration of these limitations is essential to adequately describe the behaviour and spatial ecology of a species. Our results might have been compromised by: (*i*) the presence of reefs without acoustic coverage within the array; (*ii*) disproportionate receiver coverage among reefs; and (*iii*) variability in acoustic range and detection efficiency. Despite these issues, bull sharks monitored in TSV had similar levels of residency across reefs despite differences in acoustic coverage and reef size [27]. Consequently, we are confident our results are robust and representative of the study area.

Inshore estuarine regions have been identified as essential habitats for neonate and juvenile bull sharks [12,35,67], but based on our findings adults appear to be highly dependent on coral reefs. The fact that some individuals spend a significant amount of time on coral reefs from the central GBR while others appear to use this area seasonally for foraging suggest that bull sharks may play an important role as top predator in this ecosystem. Moreover, our data demonstrated that some females undertake large seasonal migrations, potentially to give birth, and thus could act as energy links in the transfer of nutrients between systems [25,34,55]. Given that inshore areas face constant anthropogenic pressures such as fishing and habitat degradation [11,36], identifying dependence on and movement corridors between nearshore and offshore habitats is essential to understanding bull shark population dynamics. Recent studies have also highlighted the need for regional conservation approaches as bull sharks regularly cross inter-state and/or national boundaries [2,34]. A better understanding of partial migrations, habitat connectivity and philopatry in large coastal predators should be a priority for developing effective management approaches.

## Supporting Information

S1 Fig Daily environmental values for the Townsville Reefs, central Great Barrier Reef. Data were obtained from Australian Institute of Marine Science weather stations located at Davies, Rib, Kelso and Dip reefs (http://data.aims.gov.au/).
(PDF)

S2 Fig Generalized Linear Model results showing factors that influenced the number of sharks detected in the Townsville Reefs. (PDF)

S3 Fig Movement trajectories of three bull sharks monitored in the central Great Barrier Reef. (PDF)

S1 Table Description of the study reefs (central Great Barrier Reef) and estimated acoustic coverage. Acoustic coverage was calculated as the difference between total and dry reef areas divided by the sum of the detection range area at each reef. This was based on the assumption that each receiver had a maximum detection range of 250 m.
(DOCX)

S2 Table Mixed Effect Model results of factors that influence bull shark (*Carcharhinus leucas*) residency (RI) in the central Great Barrier Reef. Explanatory variables included month, sex, FL: fork length (cm); Temp: water temperature (°C); Wind: wind speed (km/h); Rain: rain accumulation (mm).
(DOCX)

S1 Video Animation of bull sharks monitored in the central Great Barrier Reef. (GIF)

S2 Video Animation of bull sharks monitored along the East coast of Australia. (GIF)

## References

[1] Heupel MR, Hueter RE. Use of an automated acoustic telemetry system to passively track juvenile blacktip shark movements In: Sibert J, Nielsen J, editors. Electronic Tagging and Tracking in Marine Fisheries: Proceedings. Dordrecht: Kluwer Academic Publishers; 2001 pp. 217–236.

[2] Daly R, Smale MJ, Cowley PD, Froneman PW. Residency patterns and migration dynamics of adult bull sharks (*Carcharhinus leucas*) on the east coast of southern Africa. PLoS One. 2014;9: e109357 10.1371/journal.pone.0109357 25295972 PMC4190266

[3] Sims DW, Southall EJ, Richardson AJ, Reid PC, Metcalfe JD. Seasonal movements and behaviour of basking sharks from archival tagging: no evidence of winter hibernation. Mar Ecol Prog Ser. 2003;248: 187–196.

[4] Carlisle AB, Kim SL, Semmens BX, Madigan DJ, Jorgensen SJ, Perle CR, et al Using stable isotope analysis to understand the migration and trophic ecology of Northeastern Pacific white sharks (*Carcharodon carcharias*). PLoS One. 2012;7: 1–15. 10.1371/journal.pone.0030492 PMC3280240 22355313

[5] Dicken M, Booth AJ, Smale M. Preliminary observations of tag shedding, tag reporting, tag wounds, and tag biofouling for raggedtooth sharks (*Carcharias taurus*) tagged off the east coast of South Africa. ICES J Mar Sci. 2006;63: 1640–1648.

[6] Dicken ML, Booth AJ, Smale MJ, Cliff G. Spatial and seasonal distribution patterns of juvenile and adult raggedtooth sharks (Carcharias taurus) tagged off the east coast of South Africa. Mar Freshw Res. 2007;58: 127.

[7] Alerstam T, Hedenstrom A, Akersson S. Long-distance migration: evolution and determinants. Oikos. 2003;103: 247–260.

[8] Olds AD, Connolly RM, Pitt KA, Maxwell PS. Habitat connectivity improves reserve performance. Conserv Lett. 2012;5: 56–63. 10.1111/j.1755-263X.2011.00204.x

[9] Frisk MG, Jordaan A, Miller TJ. Moving beyond the current paradigm in marine population connectivity: are adults the missing link? Fish Fish. 2014;15: 242–254. 10.1111/faf.12014

[10] Heupel MR, Simpfendorfer CA, Olsen EM, Moland E. Consistent movement traits indicative of innate behavior in neonate sharks. J Exp Mar Bio Ecol. Elsevier B.V.; 2012;432–433: 131–137. 10.1016/j.jembe.2012.07.013

[11] Curtis TH, Parkyn DC, Burgess GH. Use of human-altered habitats by bull sharks in a florida nursery area use of human-altered habitats. Mar Coast Fish Dyn Manag Ecosyst Sci. 2013;5: 28–38.

[12] Heupel MR, Simpfendorfer CA. Movement and distribution of young bull sharks *Carcharhinus leucas* in a variable estuarine environment. Aquat Biol. 2008;1: 277–289. 10.3354/ab00030

[13] Speed C, Field I, Meekan M, Bradshaw C. Complexities of coastal shark movements and their implications for management. Mar Ecol Prog Ser. 2010;408: 275–293. 10.3354/meps08581

[14] Chapman DD, Babcock EA, Gruber SH, DiBattista JD, Franks BR, Kessel ST, et al Long-term natal site-fidelity by immature lemon sharks (*Negaprion brevirostris*) at a subtropical island. Mol Ecol. 2009;18: 3500–3507. 10.1111/j.1365-294X.2009.04289.x 19659480

[15] Keeney DB, Heupel MR, Hueter RE, Heist EJ. Microsatellite and mitochondrial DNA analyses of the genetic structure of blacktip shark (*Carcharhinus limbatus*) nurseries in the northwestern Atlantic, Gulf of Mexico, and Caribbean Sea. Mol Ecol. 2005;14: 1911–23. 10.1111/j.1365-294X.2005.02549.x 15910315

[16] Hueter RE, Heupel MR, Heist EJ, Keeney DB. Evidence of philopatry in sharks and implications for the management of shark fisheries. J Northwest Atl Fish Soc. 2005;35: 239–247. 10.2960/J.v35.m493

[17] Chapman DD, Feldheim KA, Papastamatiou YP, Hueter RE. There and back again : a review of residency and return migrations in sharks, with implications for population structure and management. Ann Rev Mar Sci. 2015;7: 547–570. 10.1146/annurev-marine-010814-015730 25251267

[18] Papastamatiou YP, Meyer CG, Carvalho F, Dale JJ, Hutchinson MR, Holland KN. Telemetry and random-walk models reveal complex patterns of partial migration in a large marine predator. Ecology. 2013;94: 2595–606. 24400511 10.1890/12-2014.1

[19] Lea JSE, Wetherbee BM, Queiroz N, Burnie N, Aming C, Sousa LL, et al Repeated, long-distance migrations by a philopatric predator targeting highly contrasting ecosystems. Sci Rep. Nature Publishing Group; 2015;5: 11202 10.1038/srep11202 PMC4460898 26057337

[20] Chapman B, Skov C, Hulthén K, Brodersen J, Nilsson P, Hansson L, et al Partial migration in fishes: definitions, methodologies and taxonomic distribution. J Fish Biol. 2012;81: 479–499. 10.1111/j.1095-8649.2012.03349.x 22803721

[21] Espinoza M, Cappo M, Heupel MR, Tobin AJ, Simpfendorfer CA. Quantifying shark distribution patterns and species-habitat associations: implications of Marine Park Zoning. Fulton CJ, editor. PLoS One. 2014;9: e106885 10.1371/journal.pone.0106885 25207545 PMC4160204

[22] Wilson SK, Graham N a. J, Pratchett MS, Jones GP, Polunin NVC. Multiple disturbances and the global degradation of coral reefs: are reef fishes at risk or resilient? Glob Chang Biol. 2006;12: 2220–2234. 10.1111/j.1365-2486.2006.01252.x

[23] Sandin SA, Smith JE, Demartini EE, Dinsdale EA, Donner SD, Friedlander AM, et al Baselines and degradation of coral reefs in the Northern Line Islands. PLoS One. 2008;3: e1548 10.1371/journal.pone.0001548 18301734 PMC2244711

[24] Heithaus MR, Alcoverro T, Arthur R, Burkholder DA, Coates KA, Christianen MJ, et al Seagrasses in the age of sea turtle conservation and shark overfishing. Front Mar Sci. 2014;1: 1–6. 10.3389/fmars.2014.00028

[25] McCauley DJ, Young HS, Dunbar RB, Estes JA, Semmens BX, Micheli F. Assessing the effects of large mobile predators on ecosystem connectivity. Ecol Appl. 2012;22: 1711–1717. 23092009 10.1890/11-1653.1

[26] Knip DM, Heupel MR, Simpfendorfer CA. Evaluating marine protected areas for the conservation of tropical coastal sharks. Biol Conserv. Elsevier Ltd; 2012;148: 200–209. 10.1016/j.biocon.2012.01.008

[27] Espinoza M, Lédée EJ, Simpfendorfer CA, Tobin AJ, Heupel MR. Contrasting movements and connectivity of reef-associated sharks using acoustic telemetry: implications for management. Ecol Appl. 2015; 10.1890/14-2293.1 26910942

[28] Heupel MR, Semmens J, Hobday A. Automated acoustic tracking of aquatic animals: scales, design and deployment of listening station arrays. Mar Freshw Res. 2006;57: 1–13.

[29] Espinoza M, Farrugia TJ, Webber DM, Smith F, Lowe CG. Testing a new acoustic telemetry technique to quantify long-term, fine-scale movements of aquatic animals. Fish Res. 2011;108: 364–371. 10.1016/j.fishres.2011.01.011

[30] Carlson JK, Ribera MM, Conrath CL, Heupel MR, Burgess GH. Habitat use and movement patterns of bull sharks *Carcharhinus leucas* determined using pop-up satellite archival tags. J Fish Biol. 2010;77: 661–675. 10.1111/j.1095-8649.2010.02707.x 20701646

[31] IMOS. Australian Animal Tagging and Monitoring System (AATAMS) [Internet]. 2009. Available: http://imos.org.au/aatams.html

[32] Cooke SJ, Iverson SJ, Stokesbury MJW, Hinch SG, Fisk AT, VanderZwaag DL, et al Ocean tracking network Canada: a network approach to addressing critical issues in fisheries and resource management with implications for ocean governance. Fisheries. 2011;36: 583–592. 10.1080/03632415.2011.633464

[33] Hussey NE, Kessel ST, Aarestrup K, Cooke SJ, Cowley PD, Fisk AT, et al Aquatic animal telemetry: a panoramic window into the underwater world. Science (80-). 10.1126/science.1255642 26068859

[34] Heupel MR, Simpfendorfer CA, Espinoza M, Smoothy AF, Tobin AJ, Peddemors VM. Conservation challenges of sharks with continental scale migrations. Front Mar Sci. 2015;2 10.3389/fmars.2015.00012

[35] Matich P, Heithaus MR. Multi-tissue stable isotope analysis and acoustic telemetry reveal seasonal variability in the trophic interactions of juvenile bull sharks in a coastal estuary. J Anim Ecol. 2013;83: 199–213. 10.1111/1365-2656.12106 23927728

[36] Werry JM, Lee SY, Lemckert CJ, Otway NM. Natural or artificial? habitat-use by the bull shark, Carcharhinus leucas. PLoS One. 2012;7: e49796 10.1371/journal.pone.0049796 23166772 PMC3500329

[37] Curtis TH, Adams DH, Burgess GH. Seasonal distribution and habitat associations of bull sharks in the Indian River Lagoon, Florida: a 30-year synthesis. Trans Am Fish Soc. 2011;140: 1213–1226. 10.1080/00028487.2011.618352

[38] Brunnschweiler JM, Queiroz N, Sims DW. Oceans apart? Short-term movements and behaviour of adult bull sharks *Carcharhinus leucas* in Atlantic and Pacific Oceans determined from pop-off satellite archival tagging. J Fish Biol. 2010;77: 1343–1358. 10.1111/j.1095-8649.2010.02757.x 21039509

[39] Hammerschlag N, Luo J, Irschick DJ, Ault JS. A comparison of spatial andmovement patterns between sympatric predators: bull sharks (*Carcharhinus leucas*) and Atlantic tarpon (*Megalops atlanticus*). PLoS One. 2012;7: e45958. 23049904 10.1371/journal.pone.0045958 PMC3458817

[40] Done TJ. Patterns in the distribution of coral communities across the central Great Barrier Reef. Coral Reefs. 1982;1: 95–107.

[41] Espinoza M, Heupel MR, Tobin AJ, Simpfendorfer CA. Residency patterns and movements of grey reef sharks (*Carcharhinus amblyrhynchos*) in semi-isolated coral reef habitats. Mar Biol. 2015;162: 343–358.

[42] Welsh JQ, Fox RJ, Webber DM, Bellwood DR. Performance of remote acoustic receivers within a coral reef habitat: implications for array design. Coral Reefs. 2012;31: 693–702. 10.1007/s00338-012-0892-1

[43] Simpfendorfer CA, Heupel MR, Collins AB. Variation in the performance of acoustic receivers and its implication for positioning algorithms in a riverine setting. Can J Fish Aquat Sci. 2008;65: 482–492. 10.1139/F07-180

[44] Espinoza M, Heupel MR, Tobin AJ, Simpfendorfer CA. Movement patterns of silvertip sharks (Carcharhinus albimarginatus) on coral reefs. Coral Reefs. 2015;34: 807–821.

[45] Baayen RH, Davidson DJ, Bates DM. Mixed-effects modeling with crossed random effects for subjects and items. J Mem Lang. Elsevier Inc.; 2008;59: 390–412. 10.1016/j.jml.2007.12.005

[46] Zuur AF, Ieno EN, Walker N, Saveliev AA, Smith GM. Mixed Effects Models and Extensions in Ecology with R. New York, NY: Springer; 2009.

[47] Bates D, Maechler M, Bolker BM, Walker S. lme4: Linear mixed-effects models using Eigen and S4. R package version 1.0–6. http://cran.r-project.org/package=lme4 [Internet]. 2014. Report No.: R package version 1.0–6. Available: http://cran.r-project.org/package=lme4

[48] R Development Core Team. R: a language and environment for statistical computing. Vienna, Austria: R Foundationfor Statistical Computing; 2014.

[49] Simpfendorfer CA, Heupel MR, Hueter RE. Estimation of short-term centers of activity from an array of omnidirectional hydrophones and its use in studying animal movements. Can J Fish Aquat Sci. 2002;59: 23–32. 10.1139/F01-191

[50] Gu Z, Gu L, Ellis R, Schlesner M, Brors B. Circlize implements and enhances circular visualization in R. Bioinformatics. 2014;30: 2811–2812. 10.1093/bioinformatics/btu393 24930139

[51] Werry JM. Habitat ecology of the bull shark, *Carcharhinus leucas*, on urban coasts in eastern Queensland, Australia. Griffith University Gold Coast 2010.

[52] Meyer CG, Papastamatiou YP, Holland KN. A multiple instrument approach to quantifying the movement patterns and habitat use of tiger (*Galeocerdo cuvier*) and Galapagos sharks (*Carcharhinus galapagensis*) at French Frigate Shoals, Hawaii. Mar Biol. 2010;157: 1857–1868.

[53] Brunnschweiler JM, Barnett A. Opportunistic visitors: long-term behavioural response of bull sharks to food provisioning in Fiji. PLoS One. 2013;8: e58522 10.1371/journal.pone.0058522 23516496 PMC3596312

[54] Fitzpatrick R, Thums M, Bell I, Meekan MG, Stevens JD, Barnett A. A comparison of the seasonal movements of tiger sharks and green turtles provides insight into their predator-prey relationship. PLoS One. 2012;7: e51927 10.1371/journal.pone.0051927 23284819 PMC3526478

[55] Ketchum JT, Hearn A, Klimley AP, Peñaherrera C, Espinoza E, Bessudo S, et al Inter-island movements of scalloped hammerhead sharks (*Sphyrna lewini*) and seasonal connectivity in a marine protected area of the eastern tropical Pacific. Mar Biol. 2014;161: 939–951.

[56] Heupel MR, Simpfendorfer CA. Importance of environmental and biological drivers in the presence and space use of a reef-associated shark. Mar Ecol Prog Ser. 2014;496: 47–57.

[57] Vianna GM, Meekan MG, Meeuwig JJ, Speed CW. Environmental influences on patterns of vertical movement and site fidelity of grey Reef Sharks (*Carcharhinus amblyrhynchos*) at Aggregation Sites. PLoS One. 2013;8: e60331. 23593193 10.1371/journal.pone.0060331 PMC3622676

[58] Werry JM, Planes S, Berumen ML, Lee KA, Braun CD, Clua E. Reef-fidelity and migration of tiger sharks, *Galeocerdo cuvier*, across the Coral Sea. Klimley AP, editor. PLoS One. 2014;9: e83249 10.1371/journal.pone.0083249 24421879 PMC3885424

[59] Chapman BB, Hulthén K, Brodersen J, Nilsson PA, Skov C, Hansson LA, et al Partial migration in fishes: causes and consequences. J Fish Biol. 2012;81: 456–478. 10.1111/j.1095-8649.2012.03342.x 22803720

[60] Clark E, Schmidt V. Sharks of the central gulf of Florida. Bull Mar Sci. 1965;15: 13–83.

[61] Tillett BJ, Meekan MG, Field IC, Thorburn DC, Ovenden JR. Evidence for reproductive philopatry in the bull shark *Carcharhinus leucas*. J Fish Biol. 2012;80: 2140–2158. 10.1111/j.1095-8649.2012.03228.x 22551174

[62] Tobin AJ, Currey LM, Simpfendorfer CA. Informing the vulnerability of species to spawning aggregation fishing using commercial catch data. Fish Res. 2013;143: 47–56.

[63] Mapstone BD, Davies CR, Little LR, Punt AE, Smith ADM, Pantus F, et al. The effects of line fishing on the Great Barrier Reef and evaluations of alternative potential management strategies. CRC Reef Research Centre Technical Report No 52. Townsville, Australia; 2004.

[64] Heupel MR, Williams A, Welch D, Ballagh A, Mapstone B, Carlos G, et al Effects of fishing on tropical reef associated shark populations on the Great Barrier Reef. Fish Res. 2009;95: 350–361. 10.1016/j.fishres.2008.10.005

[65] Ault JS. Silver King: A most perfect and ancient sportfish—the biology, ecology, management of *Megalops atlanticus*–and its precarious future In: Mill A, editor. 2A Passion for Tarpon. Mill Creek, WA: Wild River Press; 2010 pp. 266–292.

[66] Chapman DD, Pikitch EK, Babcock E, Shivji MS. Marine reserve design and evaluation using automated acoustic telemetry: a case-study involving coral reef-associated sharks in the Mesoamerican Caribbean. Mar Technol Soc J. 2005;39: 42–55.

[67] Werry JM, Lee SY, Otway NM, Hu Y, Sumpton W. A multi-faceted approach for quantifying the estuarine—nearshore transition in the life cycle of the bull shark, Carcharhinus leucas. Mar Freshw Res. 2011;62: 1421–1431.

