# Supplementary material for: Evidence of Partial Migration in a Large Coastal Predator: Opportunistic Foraging and Reproduction as Key Drivers?
Source: PLoS One. 2016 Feb 3;11(2):e0147608. doi: 10.1371/journal.pone.0147608 (PMC4740466; doi:10.1371/journal.pone.0147608)
Supplement: S1 Fig — Data were obtained from Australian Institute of Marine Science weather stations located at Davies, Rib, Kelso and Dip reefs (http://data.aims.gov.au/). (PDF) [file pone.0147608.s001.pdf]

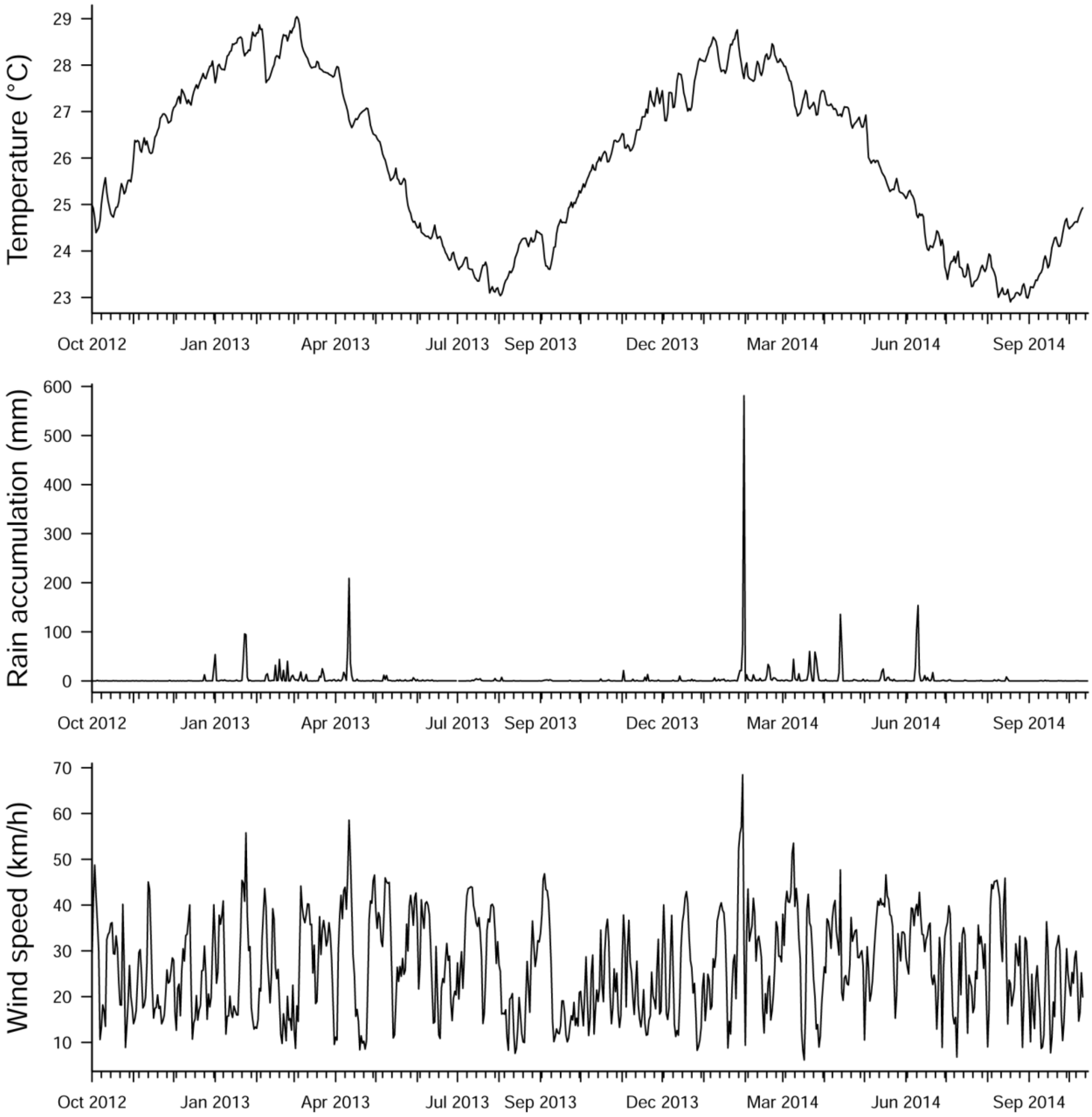

S1 Fig. Daily environmental values for the Townsville Reefs, central Great Barrier Reef. Data were obtained from Australian Institute of Marine Science weather stations located at Davies, Rib, Kelso and Dip reefs (http://data.aims.gov.au/).
