# Supplementary material for: Evidence of Partial Migration in a Large Coastal Predator: Opportunistic Foraging and Reproduction as Key Drivers?
Source: PLoS One. 2016 Feb 3;11(2):e0147608. doi: 10.1371/journal.pone.0147608 (PMC4740466; doi:10.1371/journal.pone.0147608)
Supplement: S2 Fig — (PDF) [file pone.0147608.s002.pdf]

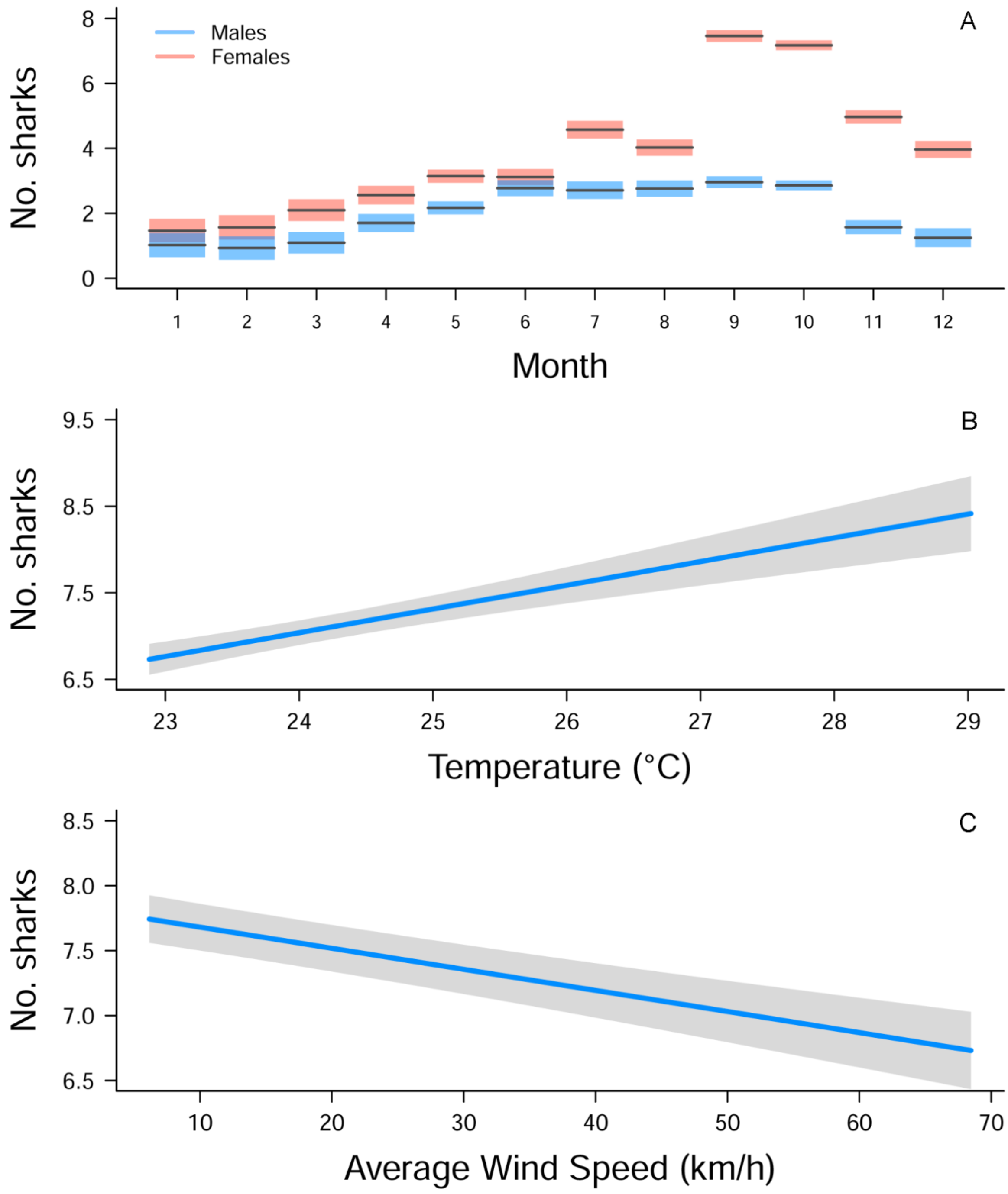

S2 Fig. Generalized Linear Model results showing factors that influenced the number of sharks detected in the Townsville Reefs.
