# Supplementary material for: Evidence of Partial Migration in a Large Coastal Predator: Opportunistic Foraging and Reproduction as Key Drivers?
Source: PLoS One. 2016 Feb 3;11(2):e0147608. doi: 10.1371/journal.pone.0147608 (PMC4740466; doi:10.1371/journal.pone.0147608)
Supplement: S3 Fig — (PDF) [file pone.0147608.s003.pdf]

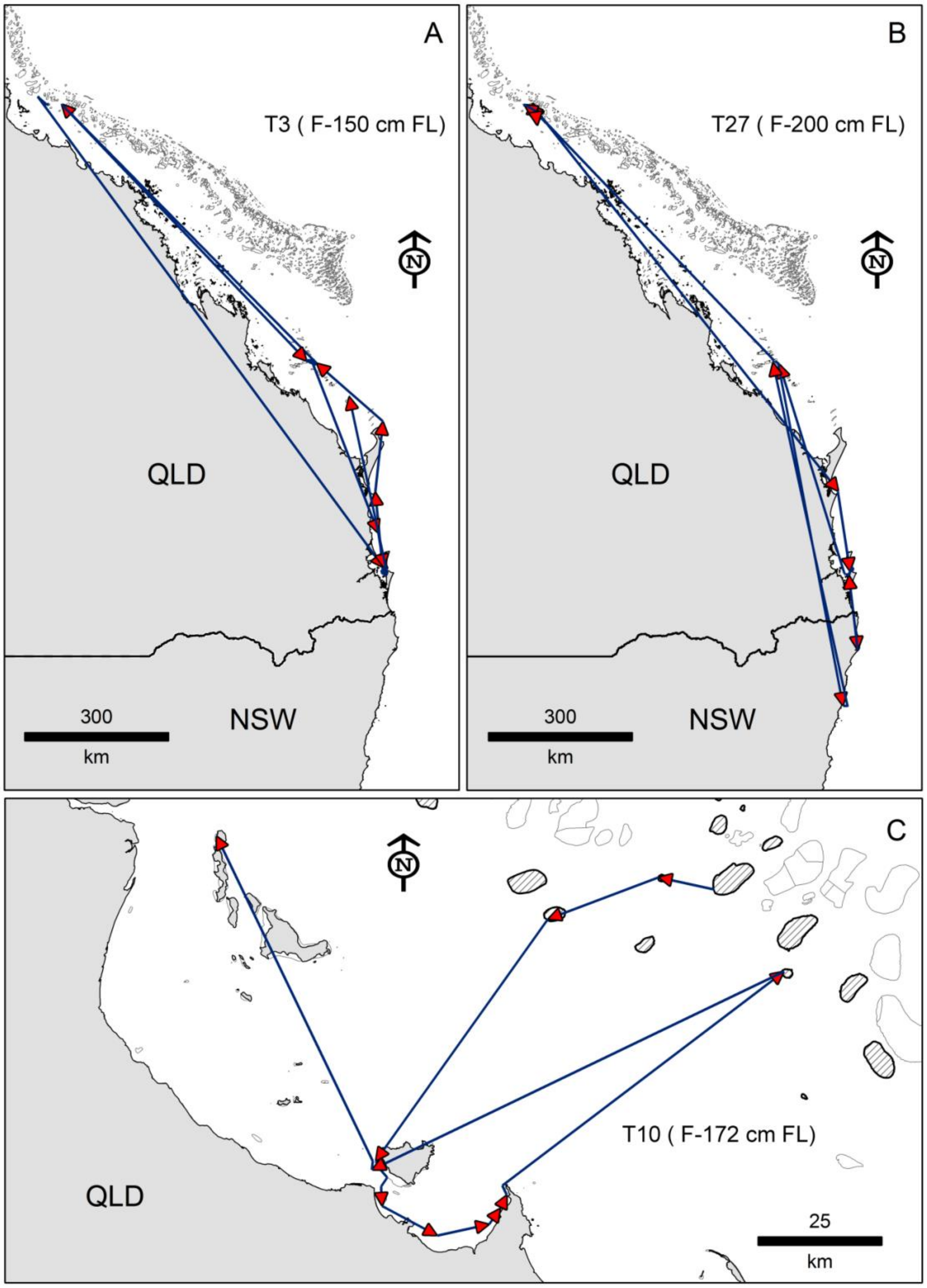

Fig S3. Movement trajectories of three bull sharks (*Carcharhinus leucas*) monitored in the central Great Barrier Reef.
