# Supplementary material for: Evidence of Partial Migration in a Large Coastal Predator: Opportunistic Foraging and Reproduction as Key Drivers?
Source: PLoS One. 2016 Feb 3;11(2):e0147608. doi: 10.1371/journal.pone.0147608 (PMC4740466; doi:10.1371/journal.pone.0147608)
Supplement: S1 Table — Acoustic coverage was calculated as the difference between total and dry reef areas divided by the sum of the detection range area at each reef. This was based on the assumption that each receiver had a maximum detection range of 250 m. (DOCX) [file pone.0147608.s004.docx]

S1 Table. Description of the study reefs (central Great Barrier Reef) and estimated acoustic coverage. Acoustic coverage was calculated as the difference between total and dry reef areas divided by the sum of the detection range area at each reef. This was based on the assumption that each receiver had a maximum detection range of 250 m.

| Reef | Zoning | Perimeter  (km) | Total reef  area (km^2^) | Dry reef  area (km^2^) | Dist. shore  (km) | Number of  Receivers | Acoustic  coverage (%) |
| --- | --- | --- | --- | --- | --- | --- | --- |
| Helix | Marine National Park | 4.5 | 1.6 | 0.4 | 68.5 | 8 | >100.0 |
| Wheeler | Marine National Park | 6.6 | 2.9 | 1.4 | 57.5 | 5 | 94.1 |
| Arc | Preservation | 3.1 | 0.6 | 0.1 | 88.3 | 1 | 56.5 |
| Lodestone | Habitat Protection | 11 | 8.7 | 4.1 | 55.2 | 8 | 49.1 |
| Pinnacle | General Use | 3.3 | 0.6 | - | 35.5 | 1 | 47.1 |
| Cotton Shoal | Preservation | 3.5 | 0.8 | - | 87.7 | 1 | 35.3 |
| Rib | Habitat Protection | 12 | 10.4 | 2.5 | 56.2 | 4 | 14.3 |
| Glow | Marine National Park | 11.2 | 8.8 | 0.8 | 83.1 | 3 | 14.1 |
| Keeper | Habitat Protection | 10.7 | 7.2 | 2.9 | 55.1 | 2 | 13.1 |
| Yankee | Marine National Park | 10.2 | 6.7 | 1.1 | 82.7 | 3 | 10.1 |
| John Brewer | Conservation Park | 18.9 | 24.6 | 5.5 | 61.1 | 4 | 7.4 |
| Kelso | Marine National Park | 15.5 | 12.8 | 1.9 | 69.4 | 2 | 5.2 |
| Davies | Conservation Park | 17.1 | 16.6 | 2.9 | 59.4 | 2 | 4.1 |
| Grub | Habitat Protection | 22.5 | 31.3 | 2.5 | 74.9 | 4 | 3.9 |
| Broadhurst | Habitat Protection | 20.3 | 27.1 | 2 | 49.3 | 2 | 2.3 |
| Centipede | Habitat Protection | 23.3 | 30.1 | 2.9 | 66.1 | 2 | 2.1 |
| Bramble | Habitat Protection | 30.7 | 62.9 | 8.5 | 41 | 4 | 2.1 |
