# Supplementary material for: Evidence of Partial Migration in a Large Coastal Predator: Opportunistic Foraging and Reproduction as Key Drivers?
Source: PLoS One. 2016 Feb 3;11(2):e0147608. doi: 10.1371/journal.pone.0147608 (PMC4740466; doi:10.1371/journal.pone.0147608)
Supplement: S2 Table — Explanatory variables included month, sex, FL: fork length (cm); Temp: water temperature (°C); Wind: wind speed (km/h); Rain: rain accumulation (mm). (DOCX) [file pone.0147608.s005.docx]

S2 Table. Mixed Effect Model results of factors that influence bull shark (*Carcharhinus leucas*) residency (RI) in the central Great Barrier Reef. Explanatory variables included month, sex, FL: fork length (cm); Temp: water temperature (°C); Wind: wind speed (km/h); Rain: rain accumulation (mm).

| Rank | Model | df | AICc | p-value |
| --- | --- | --- | --- | --- |
| 1 | RI ~ Month × Sex | 26 | 4651.8 | <0.001^a^ |
| 2 | RI ~ Month × FL | 26 | 4726.7 | <0.001^a^ |
| 3 | RI ~ Month | 14 | 4795.2 | <0.001^b^ |
| 4 | RI ~ Month + FL | 15 | 4796.6 | <0.001^b^ |
| 5 | RI ~ Month + Sex | 15 | 4797.1 | <0.001^b^ |
| 6 | RI ~ Month + Sex + FL | 16 | 4798.6 | <0.001^b^ |
| 7 | RI ~ Month + Sex × FL | 17 | 4800.2 | <0.001^b^ |
| 8 | RI ~ Rain + Wind | 5 | 5064.3 | <0.001^b^ |
| 9 | RI ~ Temp + Rain + Wind | 6 | 5064.9 | <0.001^b^ |
| 10 | RI ~ Temp + Wind | 5 | 5077.7 | <0.001^b^ |
| 11 | RI ~ Wind | 4 | 5089.2 | <0.001^b^ |
| 12 | RI ~ FL + Wind | 5 | 5090.7 | <0.001^b^ |
| 13 | RI ~ Sex + Wind | 5 | 5091.2 | <0.001^b^ |
| 14 | RI ~ Sex + FL + Wind | 6 | 5092.6 | <0.001^b^ |
| 15 | RI ~ Sex × FL + Wind | 7 | 5094.2 | <0.001^b^ |
| 16 | RI ~ Temp + Rain | 5 | 5422.4 | <0.001^b^ |
| 17 | RI ~ Rain | 4 | 5423.0 | <0.001^b^ |
| 18 | RI ~ Temp + Rain | 5 | 5425.0 | <0.001^b^ |
| 19 | RI ~ Sex × FL + Rain | 6 | 5426.4 | <0.001^b^ |
| 20 | RI ~ Sex × FL + Rain | 7 | 5428.1 | <0.001^b^ |
| 21 | RI ~ FL + Rain | 5 | 5524.5 | <0.001^b^ |
| 22 | RI ~ Temp | 4 | 5631.7 | <0.001^b^ |
| 23 | RI ~ FL + Temp | 5 | 5633.2 | <0.001^b^ |
| 24 | RI ~ Sex + Temp | 5 | 5633.7 | <0.001^b^ |
| 25 | RI ~ Sex + FL + Temp | 6 | 5635.6 | <0.001^b^ |
| 26 | RI ~ Sex × FL + Temp | 7 | 5636.8 | <0.001^b^ |
| 27 | RI ~ FL | 4 | 5679.6 | 0.478^c^ |
| 28 | RI ~ Sex × FL | 6 | 5683.2 | 0.823^c^ |
| 29 | RI ~ Sex | 4 | 5697.7 | 0.847^c^ |

Significant differences of candidate models from the null model [RI ~ 1 + (1 | Tag ID) + (1 | Year)] were evaluated with maximum likelihood ratio tests (χ^2^, p < 0.05). Top two models were similar to each other but statistically different from the rest (letters a and b).
