# Supplementary figures and images for: Evidence of Partial Migration in a Large Coastal Predator: Opportunistic Foraging and Reproduction as Key Drivers?
Source: PLoS One. 2016 Feb 3;11(2):e0147608. doi: 10.1371/journal.pone.0147608 (PMC4740466; doi:10.1371/journal.pone.0147608)

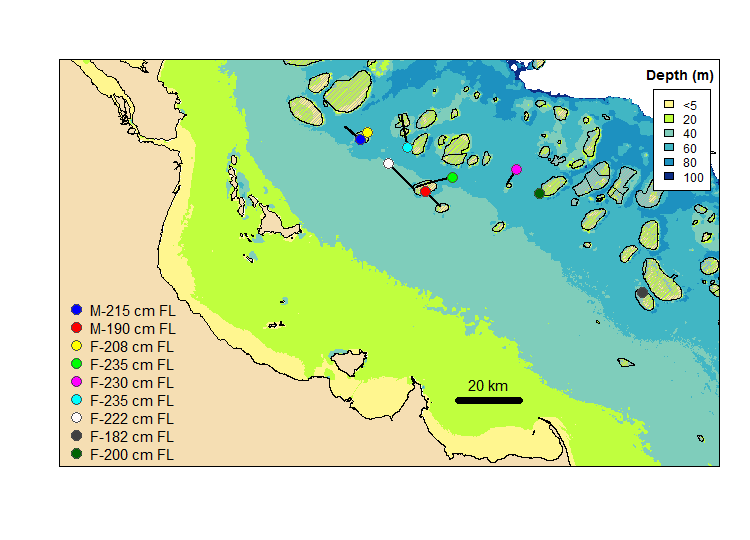

Supplement: S1 Video — (GIF) [file pone.0147608.s006.gif]

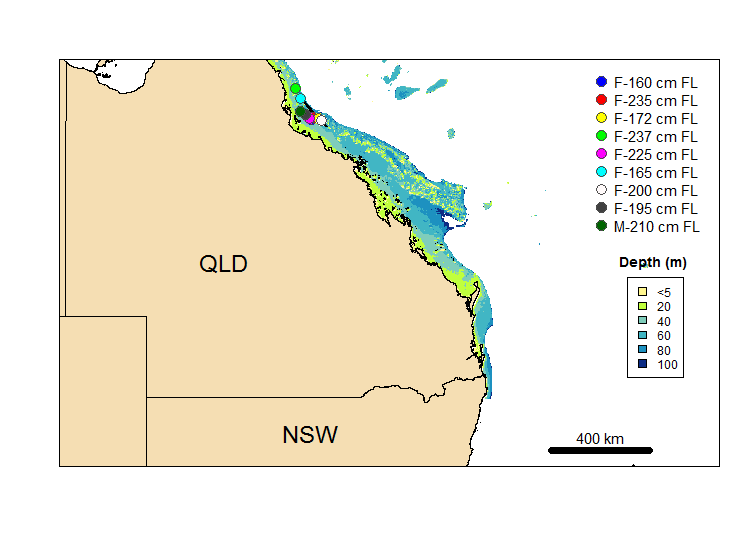

Supplement: S2 Video — (GIF) [file pone.0147608.s007.gif]
